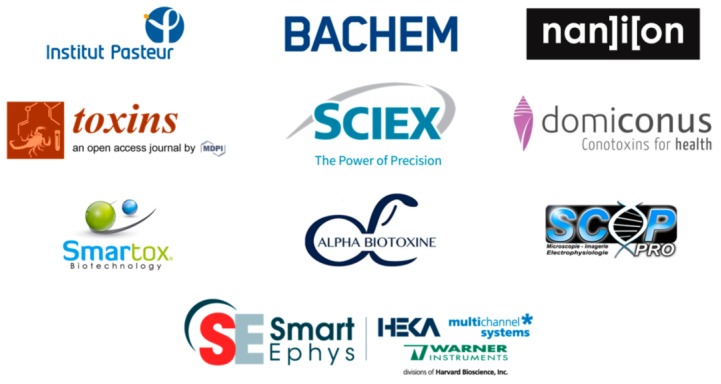# Report from the 26th Meeting on Toxinology, “Bioengineering of Toxins”, Organized by the French Society of Toxinology (SFET) and Held in Paris, France, 4–5 December 2019

**DOI:** 10.3390/toxins12010031

**Published:** 2020-01-03

**Authors:** Pascale Marchot, Sylvie Diochot, Michel R. Popoff, Evelyne Benoit

**Affiliations:** 1 Laboratoire ‘Architecture et Fonction des Macromolécules Biologiques’, CNRS/Aix-Marseille Université, Faculté des Sciences-Campus Luminy, 13288 Marseille CEDEX 09, France; 2 Institut de Pharmacologie Moléculaire et Cellulaire, Université Côte d’Azur, CNRS, Sophia Antipolis, 06550 Valbonne, France; diochot@ipmc.cnrs.fr; 3 Bacterial Toxins, Institut Pasteur, 75015 Paris, France; michel-robert.popoff@pasteur.fr; 4 Service d’Ingénierie Moléculaire des Protéines (SIMOPRO), CEA de Saclay, Université Paris-Saclay, 91191 Gif-sur-Yvette, France

## 1. Preface

This 26th edition of the annual Meeting on Toxinology (RT26) of the SFET (http://sfet.asso.fr/international) was held at the Institut Pasteur of Paris on 4–5 December 2019. The central theme selected for this meeting, “Bioengineering of Toxins”, gave rise to two thematic sessions: one on animal and plant toxins (one of our “core” themes), and a second one on bacterial toxins in honour of Dr. Michel R. Popoff (Institut Pasteur, Paris, France), both sessions being aimed at emphasizing the latest findings on their respective topics. Nine speakers from eight countries (Belgium, Denmark, France, Germany, Russia, Singapore, the United Kingdom, and the United States of America) were invited as international experts to present their work, and other researchers and students presented theirs through 23 shorter lectures and 27 posters. Of the ~80 participants who registered, ~40% were foreigners (Algeria, Belgium, Denmark, France, Germany, Italy, the Netherlands, Russia, Singapore, the United Kingdom, and the United States of America), thereby highlighting the international attractiveness of the SFET meetings. For this RT26, the SFET aimed to ensure a fair balance between participants interested in toxins from the animal/plant versus bacterial kingdoms.

Owing to a donation from MDPI *Toxins*, two prizes of 250 EUR each were awarded to the best oral communication and the best poster, both selected by a jury of 10 persons. Various useful or amusing goodies, generously offered by our sponsors, were distributed to all presenters.

Last but not least, we warmly thank the Editors of MDPI *Toxins* for permitting the publication of a Special Issue focused on the “Bioengineering of Toxins” and gathering this meeting report, along with peer-reviewed original articles and reviews. We hope that this Special Issue will be attractive to all, including those colleagues who could not attend the RT26 meeting, and that it will represent a comprehensive source of information for researchers and students in the field of Toxinology.


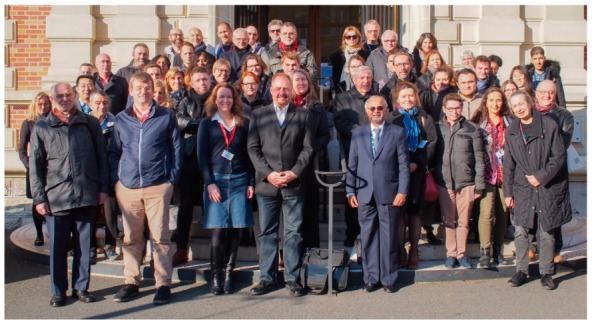
 *Missing in the picture: Julien Barbier, Daniel Gillet, Daniel Ladant, Pascale Marchot, and perhaps a few others*

## 2. Scientific and Organizing Committee (SFET Board of Directors)

Julien Barbier, CEA de Saclay, Gif-sur-Yvette, France;
Evelyne Benoit, CEA de Saclay, Gif-sur-Yvette, France;
Alexandre Chenal, Institut Pasteur, Paris, France;
Michel De Waard, Institut du Thorax, Nantes, France;
Sylvie Diochot, Institut de Pharmacologie Moléculaire et Cellulaire, Valbonne, France;
Sébastien Dutertre, Institut des Biomolécules Max Mousseron, Montpellier, France;
Daniel Ladant, Institut Pasteur, Paris, France;
Christian Legros, Université d’Angers, Angers, France;
Pascale Marchot, CNRS/Aix-Marseille Université, Marseille, France;
Gilles Prévost, Institut de Bactériologie, Université de Strasbourg, France;
Michel R. Popoff, Institut Pasteur, Paris, France;
Loïc Quinton, Université de Liège, Liège, Belgium.

## 3. Invited Lectures (When More Than One Author, the Underlined Name Is That of the Presenter)

### 3.1. Characterization of a Membrane-Binding Loop Leads to Engineering Botulinum Neurotoxin B with Enhanced Efficacy for Targeting Neurons

Linxiang Yin ^1^, Geoffrey Masuyer ^2^, Sicai Zhang ^1^, Jie Zhang ^1^, Shin-Ichiro Miyashita ^1^, David Burgin ^3^, Laura Lovelock ^3^, Shu-Fen Coker ^3^, Tian-Min Fu ^4^, Matthew Beard ^3^, Pål Stenmark ^2,5^ and Min Dong^1,^*

^1^  Department of Urology, Boston Children’s Hospital, Department of Microbiology and Immunobiology and Department of Surgery, Harvard Medical School, Boston, MA 02115, USA
^2^  Department of Biochemistry and Biophysics, Stockholm University, 10691 Stockholm, Sweden
^3^  Ipsen Bioinnovation, 102 Park Drive, Milton Park, Abingdon OX14 4RY, UK
^4^  Department of Biological Chemistry and Molecular Pharmacology, Harvard Medical School, Program in Cellular and Molecular Medicine, Boston Children’s Hospital, Boston, MA 02115, USA
^5^  Department of Experimental Medical Science, Lund University, 22100 Lund, Sweden

* Correspondence: min.dong@childrens.harvard.edu

**Abstract:** Botulinum neurotoxins are a family of bacterial toxins with seven major serotypes (BoNT/A–G). They target motor nerve terminals with extreme specificity by recognizing gangliosides and synaptic vesicle proteins as dual receptors. Recent studies suggest that an extended loop region in the receptor-binding domain (HC) in BoNT/B, C, D, G, and a chimeric DC toxin may also contribute to toxin binding to neurons by interacting with lipid membranes (termed lipid-binding loop, LBL). Here, we characterize the binding of toxins to receptor-free lipid membranes and ganglioside/protein receptors embedded in the lipid environment, utilizing nanodisc technology and biolayer interferometry assays. We find that the LBLs of BoNT/DC, C and G, but not the LBLs of BoNT/B and D, can bind to lipid membranes. Mutagenesis studies at the tip of BoNT/DC-LBL demonstrate the critical role of aromatic residues in mediating lipid membrane interactions. By replacing two residues at the tip of BoNT/B-LBL with aromatic residue tryptophan, we create a “gain-of-function” mutant (HC/BWW) that can bind lipid membranes and shows enhanced binding to cultured neurons compared to HC/B. Co-crystallization studies confirm that the two tryptophan residues are located at the tip of BoNT/B-LBL and do not alter the structure of HC/B or interactions with its receptors. Full-length active BoNT/B containing HC/BWW is produced and evaluated in mice in vivo using Digit Abduction Score assays. This mutant toxin shows enhanced efficacy in paralyzing local muscles at the injection site and reduced systemic diffusion, thus extending both its safety range and the duration of paralysis compared with the control BoNT/B. These findings establish a mechanistic understanding of LBL–lipid interactions and create an engineered BoNT/B with improved therapeutic efficacy.

**Keywords:** botox; botulinum neurotoxin; lipid binding; receptor

### 3.2. From Toxins to Therapeutics: New Cardiovascular Therapeutic Agents

Manjunatha Kini *

Postal Department of Biological Sciences, Faculty of Science, National University of Singapore, 117543 Singapore, Singapore

* Correspondence: dbskinim@nus.edu.sgx

**Abstract:** Cardiovascular diseases are the major cause of death in both the developed and developing world. With an aging population and changing lifestyles, cardiovascular diseases are on the raise. Despite bypass surgeries, balloon angioplasty, cardiovascular resuscitation, and various preventive strategies, death and debilitation due to cardiovascular diseases are substantial. Hence, novel therapeutic agents are sought after to reduce mortality and morbidity. Animal venoms and the saliva of blood-feeding animals are excellent sources of bioactive molecules, and they provide opportunities to develop novel therapeutic agents to treat various diseases. Here, I will describe the development of (a) a novel parenteral anticoagulant for balloon angioplasty based on a new class of thrombin inhibitors from tick saliva and (b) two human natriuretic peptide analogues for the treatment of heart failure patients. DRD-ANP induces only vasodilatory effects without diuretic function, while DGD-ANP induces only diuretic effects without vasodilatory function. The diuretic and hemodynamic effects of these human ANP analogues were evaluated in anesthetized rat models as well as conscious normal and heart failure sheep models. These distinct classes of NPAs will be useful in the treatment of distinct subtypes of acute decompensated heart failure (ADHF) patients.

**Keywords:** drug design; krait venom; percutaneous coronary intervention; tick salivary protein

### 3.3. Discovery of Monoclonal Antibodies for Snakebite Envenoming Therapy and Diagnosis

Cecilie Knudsen ^1,2,^*, Søren H. Dam ^1^, Aleksander M. Haack ^1^, Rasmus U. W. Friis ^1^, Jonas A. Jürgensen ^1^, Shirin Ahmadi ^1,3^, Ana S. Arias ^4^, Urska Pus ^1^, Saioa Oscoz ^4^, Edward W. Masters ^5^, Alice M. Luther ^5^, Daniel T. Griffiths ^5^, Maiken L. Olesen ^5^, Rachael A. Leah ^5^, Peter Slavny ^5^, Manuela B. Pucca ^1,6^, Felipe A. Cerni ^1,7^, Line Ledsgaard ^1^, Christoffer V. Sørensen ^1^, Sofie Føns ^1^, Rahel Janke ^1^, Julius Knerr ^1^, Andreas Bertelsen ^1^, Rasmus I. Dehli ^1^, Andrea M. Esteban ^1^, Eliane C. Arantes ^7^, Bruno Lomonte ^4^, José. M. Gutiérrez ^4^, Aneesh Karatt-Vellatt ^5^, John McCafferty ^5^, Jan K. Andersen ^2^ and Andreas H. Laustsen ^1^

^1^  Department of Biotechnology and Biomedicine, Technical University of Denmark, 2800 Kongens Lyngby, Denmark
^2^  BioPorto Diagnostics A/S, 2900 Hellerup, Denmark
^3^  Department of Biotechnology and Biosafety, Eskişehir Osmangazi University, 26120 Eskişehir, Turkey
^4^  Instituto Clodomiro Picado, Facultad de Microbiología, Universidad de Costa Rica, 22060 San José, Costa Rica
^5^  IONTAS, Iconix Park, Pampisford, Cambridge CB22 3EG, UK
^6^  Medical School, Federal University of Roraima, Boa Vista 69310-000, Brazil
^7^  Department of Physics and Chemistry, School of Pharmaceutical Sciences of Ribeirão Preto, University of São Paulo, São Paulo 05508-220, Brazil

* Correspondence: cecknu@dtu.dk

**Abstract:** Snakebite envenoming is a neglected tropical disease, which for many years has been largely ignored by the wider scientific community, funding agencies, and policy makers. However, with the 2017 reinstatement of snakebite envenoming on the World Health Organization category A list of neglected tropical diseases, a new era might be dawning for the management of snakebite envenoming, and many novel opportunities for innovating snakebite diagnostics and therapeutics may present themselves. Here, we describe two attempts at pursuing such opportunities for novel solutions. Firstly, we describe the discovery and evaluation of 24 recombinant, fully human monoclonal immunoglobulin G (IgG) antibodies for use in a prototypic, recombinant antivenom against the neurotoxic components of black mamba (*Dendroaspis polylepis*) venom. These efforts resulted in the creation of a cocktail of three antibodies, which proved capable of providing full protection against dendrotoxin-mediated neurotoxicity of black mamba whole venom in an in vivo model of envenoming. These antibodies could either supplement existing antivenoms based on animal-derived antibodies or serve as a precursor for future recombinant antivenoms with improved safety profiles based solely on human monoclonal antibodies. The constituent antibodies of such antivenoms could be engineered to be broadly neutralising, and the preliminary results of such an engineering process will be demonstrated. Secondly, we describe the discovery and evaluation of 117 murine IgG antibodies against the venom of Brazilian snake species. These antibodies are intended for use in a diagnostic lateral flow assay. Such an assay might be able to detect and identify toxins in the blood of victims before clinical manifestations become apparent to the treating physician, thereby potentially allowing physicians to initiate treatment early on. It is also possible that the assay could be used by epidemiologists to support the mapping of snakebite incidence. Increased knowledge of incidence could help to raise awareness, improve resource management during antivenom procurement, and might guide the design of novel antivenoms.

**Keywords:** diagnostics; monoclonal antibody; snakebite envenoming; therapeutics

### 3.4. Bioengineering of *Bordetella pertussis* Adenylate Cyclase Toxin for Vaccinal and Biotechnological Purposes

Daniel Ladant *

Biochimie des Interactions Macromoléculaires, Institut Pasteur, 75015 Paris, France

* Correspondence: daniel.ladant@pasteur.fr

**Abstract:** The adenylate cyclase toxin, CyaA is an essential virulence factor from *Bordetella pertussis*, and plays a critical role in the early stages of the respiratory tract colonization by this pathogen. It invades phagocytic cells, where it is activated by calmodulin to produce supraphysiological levels of cAMP, and thus alters the phagocytic functions of these cells. CyaA is a 1706-amino-acid-long protein that belongs to the RTX toxin family of cytolysins. The main originality of this toxin resides in its unique mechanism of penetration into eukaryotic cells: after binding to target cells, CyaA is able to rapidly deliver its catalytic domain directly across the plasma membrane of the target cells. Recent progress in the understanding of this remarkable process has highlighted how the structural disorder of the CyaA toxin is leveraged to ensure its efficient secretion by bacteria and rapid invasion of target cells. I will illustrate how this flexibility has been harnessed to engineer CyaA as a molecular Trojan horse for antigen delivery into antigen-presenting cells, or to design versatile CyaA-based screening technologies in bacteria.

**Keywords:** adenylate cyclase; *Bordetella*; two-hybrid; vaccine vehicle

### 3.5. Bioengineering of Cone Snail and Spider Toxins into Small Cyclic Ion-Channel Inhibitors

Steve Peigneur^1,^*, Cristina Da Costa Oliveira ^2^, Flávia Cristina De Sousa Fonseca ^2^, Alexander Mueller ^3^, Kirsten Mcmahon ^3^, Olivier Cheneval ^3^, Anne-Sophie Depuydt ^1^, Ana Cristina Nogueira Freitas ^5^, Hana Starobova ^3^, Igor Dimitri Gama Duarte ^2^, David J. Craik ^3^, Irina Vetter ^3,4^, Maria Elena De Lima ^5,6^, Christina I. Schroeder ^3^ and Jan Tytgat ^1^

^1^  Toxicology and Pharmacology, Katholieke Universiteit (KU) Leuven, Campus Gasthuisberg, 3000 Leuven, Belgium
^2^  Department of Pharmacology, Institute of Biological Sciences, Federal University of Minas Gerais (UFMG), Belo Horizonte, Minas Gerais 31270-901, Brazil
^3^  Institute for Molecular Bioscience, The University of Queensland, St. Lucia, QLD 4072, Australia
^4^  School of Pharmacy, The University of Queensland, Woolloongabba, QLD 4102, Australia
^5^  Departamento de Bioquímica e Imunologia, Laboratório de Venenos e Toxinas Animais, Instituto de Ciências Biológicas, Universidade Federal de Minas Gerais, Belo-Horizonte 31270-901, Brazil
^6^  Programa de Pós-Graduação em Ciências da Saúde, Biomedicina e Medicina, Instituto de Ensino e Pesquisa da Santa Casa de Belo Horizonte, Grupo Santa Casa de Belo Horizonte, Belo Horizonte 30260-070, Brazil

* Correspondence: steve.peigneur@pharm.kuleuven.be

**Abstract:** Voltage-gated sodium (NaV) channels play crucial roles in a range of (patho)physiological processes. Much interest has arisen within the pharmaceutical industry in pursuing these channels as analgesic targets following the overwhelming evidence that the NaV channel subtypes NaV1.7–NaV1.9 are involved in nociception. More recently, NaV1.1, NaV1.3 and NaV1.6 have also been identified as involved in pain pathways. Venom-derived, disulfide-rich peptide toxins, isolated from spiders and cone snails, have been used extensively as probes to investigate these channels and have attracted much interest as drug leads for pharmaceutical development. However, few peptide drug leads have made it as drugs, due to their unfavourable physiochemical attributes including being poor in vivo pharmacokinetics, and having rapid proteolytic cleavage and limited oral bioavailability. The present work aims to bridge the gap in the development pipeline between drug leads and drugs candidates by downsizing these larger venom-derived NaV inhibitors into smaller, more “drug-like” molecules. As a start, a 13 amino acid, voltage-gated, sodium (NaV) channel inhibitor peptide, Pn, containing two disulfide bridges, was designed using a chimeric approach. This approach was based on a common pharmacophore deduced from the sequence and secondary structural homology of two NaV inhibitors: *Conus kinoshitai* toxin KIIIA, a 14 residue cone snail peptide with three disulfide bonds, and *Phoneutria nigriventer* toxin 1, a 78 residue spider toxin with seven disulfide bonds. As with the parent peptides, this novel NaV channel inhibitor was active on NaV1.2. Through the generation of three series of peptide mutants, we investigated the role of key residues and cyclization, and their influence on NaV inhibition and subtype selectivity. Cyclic PnCS1, a ten-residue peptide cyclized via a disulfide bond, exhibited increased inhibitory activity toward therapeutically relevant NaV channel subtypes, including NaV1.7 and NaV1.9, while displaying remarkable serum stability. Using sophisticated peptide engineering of small cyclic peptide design to aid in the determination of what drives the subtype selectivity and molecular interactions of these downsized inhibitors across NaV subtypes, we designed a series of small, stable and novel NaV probes based on PnCS1. These analogous display interesting subtype selectivity and potency in vitro, coupled with exciting in vivo analgesic activity, rendering these peptides potential analgesic drug candidates. Furthermore, we show that our design strategy can also be used to design inhibitors of voltage-gated calcium channels. These peptides represent the smallest cyclic peptidic ion channel modulators to date and are promising templates for the development of toxin-based therapeutic agents.

**Keywords:** cone snail; peptide toxin; voltage-gated sodium channel

### 3.6. Synthetic and Heterologously Expressed Toxins from Snakes, *Conus* Mollusks and Scorpions in Research on the Nicotinic Acetylcholine Receptors

Yuri Utkin *, Igor Kasheverov, Vladimir Kost, Peter Oparin, Oksana Nekrasova, Igor Ivanov, Denis Kudryavtsev, Alexander Vassilevski and Victor Tsetlin

Shemyakin-Ovchinnikov Institute of Bioorganic Chemistry RAS, 11799 Moscow, Russia

* Correspondence: utkin@mx.ibch.ru

**Abstract:** Nicotinic acetylcholine receptors (nAChRs) are targeted by a number of toxins. The best known are α-neurotoxins and α-conotoxins, from the Elapidae snakes and *Conus* mollusks, respectively. However, the multiplicity of nAChR subtypes requires the discovery of new subtype-specific ligands, and very often these compounds are present in animal venoms in extremely low amounts, insufficient for extensive study of biological activity. Larger quantities can be prepared by peptide synthesis or heterologous expression in bacteria. Our studies on the biological activity of scorpion venoms revealed their anticholinergic activity, for which the already-known toxins OSK-1 from *Orthochirus scrobiculosus*, spinoxin from *Heterometrus spinifer* and HelaTx1 from *H. laoticus* were responsible. All of them are blockers of voltage-gated potassium channels. For detailed biological activity studies, the toxins were prepared either by peptide synthesis (spinoxin and HelaTx1) or by heterologous expression in *Escherichia coli* (charybdotoxin, hongotoxin-1, kaliotoxin-1 and agitoxin-2). Investigation of these toxins revealed their micromolar and sub-micromolar affinities towards muscle-type *Torpedo* nAChR. The most active compounds (OSK-1 and spinoxin), in competition with α-bungarotoxin, showed IC_50_ of about 0.5 μM. Similar blocking efficacy was revealed in the functional test on mouse muscle-type nAChR, expressed in *Xenopus* oocytes. The affinity of all tested scorpion toxins to the human neuronal α7 receptor was significantly lower. While scorpion toxins and conotoxins possessing several disulfides require the correct closure of disulfide bonds after synthesis, a linear peptide azemiopsin from *Azemiops feae* venom is much easier to synthesize. The synthetic azemiopsin efficiently competed with α-bungarotoxin for binding to the *Torpedo* muscle-type nAChR (IC_50_ = 0.18 μM) and with lower efficiency to the human neuronal α7 nAChR (IC_50_ = 22 μM). It dose-dependently blocked acetylcholine-induced currents in *Xenopus* oocytes heterologously expressing the human muscle-type nAChR, and was more potent against the adult, ε-subunit-containing form (EC_50_ = 0.44 μM) than the fetal, γ-subunit-containing form (EC_50_ = 1.56 μM). There are numerous data about the presence of transcripts for three-finger toxins in the venom glands of Viperidae snakes. However, there are no data about the putative biological activity of viper three-finger toxins. By heterologous expression in *E. coli* we prepared two toxins, TFT-AF and TFT-VN, the amino acid sequences of which were deduced from cDNA sequences cloned from venom glands of the vipers *A. feae* and *Vipera nikolskii*, respectively. The study of their biological activity showed that the viper three-finger toxins are antagonists of neuronal as well as muscle-type nAChRs.

**Keywords:** neurotoxin; nicotinic acetylcholine receptor; scorpion; three-finger toxin; venom; viper

**Funding:** This work was supported by RFBR grant № 18-04-0107.5.

## 4. Oral Presentations (When More Than One Author, the Underlined Name Is That of the Presenter)

### 4.1. The Revisited Purifications of Various Snake Proteins of Different Sizes

Soioulata Aboudou ^1,2^, Pascal Mansuelle ^3^, Alaeddine Redissi ^2,4^, Dorine Palud ^2^, Yasmine Boughanmi ^1,2^, Harold De Pomyers ^1^, Hugues Baeza ^1^, Didier Gigmes ^2^ and Kamel Mabrouk ^2,^*

^1^  LATOXAN SAS, 845 avenue Pierre Brossolette, 26800 Portes-lès-Valence, France
^2^  Aix-Marseille Université/CNRS Institut de Chimie Radicalaire UMR 7273, 13397 Marseille CEDEX 20, France
^3^  CNRS Campus Joseph Aiguier Plateforme Protéomique FR 3479 IMM, 31 chemin Joseph Aiguier, 13402 Marseille CEDEX 20, France
^4^  Université Manouba, ISBST, BVBGR-LR11ES31, BiotechPole Sidi Thabet, 2020 Ariana, Tunisia

* Correspondence: kamel.mabrouk@univ-amu.fr

**Abstract:** Venoms are a valuable source of unique and novel chemical compounds with many potential therapeutic benefits. Their high specificity of action, their stability and their resistance to physiological inhibitors made it possible to identify them as targets of choice in diagnostic tests and as a pharmacological tool in the search for new therapeutic agents. It should be noted that these target proteins are generally weakly present in complex mixture venom. This low presence makes their purification method difficult and expensive. Indeed, in the literature, several steps are generally necessary for the purification of proteins. This multitude of steps reduces the production yield. Protein purification is a key step, and therefore it is important to optimize the methodology used. In this work, we will present several examples of protein purifications. These proteins were isolated from animal venoms and have different sizes—fasciculin 2 (6.8 kDa), α-cobratoxin (7.8 kDa), notexin (13.6 kDa), echicetin (30 kDa) and CVFm (150 kDa)—and different levels of structure complexity.

**Keywords:** protein; purification; snake venom

### 4.2. The Pore Structure of *Clostridium perfringens* Epsilon Toxin

Christos G. Savva ^1^, Alice R. Clark ^2^, Claire E. Naylor ^3^, Michel R. Popoff ^4^, David S. Moss ^5^, Ajit K. Basak ^5^, Richard W. Titball ^6^ and Monika Bokori-Brown^6,^*

^1^  Leicester Institute of Structural and Chemical Biology, Department of Molecular and Cell Biology, University of Leicester, Leicester LE1 7JA, UK
^2^  Faculty of Science and Engineering, University of Wolverhampton, Wolverhampton WV1 1AD, UK
^3^  Molecular Dimensions, Newmarket CB8 7SQ, UK
^4^  Bacterial Toxins, Institut Pasteur, 75015 Paris, France
^5^  Department of Biological Sciences, Birkbeck College, London WC1E 7HX, UK
^6^  College of Life and Environmental Sciences, University of Exeter, Exeter EX4 4ST, UK

* Correspondence: m.bokori-brown@exeter.ac.uk

**Abstract:** Epsilon toxin (Etx), a potent pore-forming toxin (PFT) produced by *Clostridium perfringens*, is responsible for the pathogenesis of enterotoxaemia of ruminants, and has been suggested to play a role in multiple sclerosis in humans. Etx is a member of the aerolysin family of β-PFTs (aβ-PFTs). While the Etx’s soluble monomer structure was solved in 2004, Etx pore structure has remained elusive, due to the difficulty of isolating the pore complex. Here, we show the cryo-electron microscopy structure of Etx pore assembled on the membrane of susceptible cells. The pore structure explains important mutant phenotypes and suggests that the double β-barrel, a common feature of the aβ-PFTs, may be an important structural element in driving efficient pore formation. These insights provide a framework for the development of novel therapeutics to prevent human and animal infections, and are relevant for nano-biotechnology applications.

**Keywords:** epsilon toxin; pore structure

### 4.3. Post-Translational Acylation and Calcium Binding Control the Folding and Activity of the *Bordetella pertussis* CyaA Toxin

Darragh P. O’Brien ^1^, Alexis Voegele ^1^, Dorothée Raoux-Barbot ^1^, Marilyne Davi ^1^, Sara Cannella ^1^, Thibaut Douche ^1^, Mariette Matondo ^1^, Dominique Durand ^2^, Patrice Vachette ^2^, Sébastien Brier ^1^, Daniel Ladant ^1^ and Alexandre Chenal^1,^*

^1^  Institut Pasteur, 75015 Paris, France
^2^  Institute for Integrative Biology of the Cell (I2BC), CEA, CNRS, Univ. Paris-Sud, Université Paris-Saclay, 91198 Gif-sur-Yvette, France

* Correspondence: chenal@pasteur.fr

**Abstract:** The contributions of post-translational modifications to the folding and activity of proteins are still poorly understood. The adenylate cyclase toxin, CyaA, is a major virulence factor of *Bordetella pertussis*, the causative agent of whooping cough, and plays an essential role in the early stages of respiratory tract colonization. CyaA is produced as an inactive pro-toxin, which is post-translationally acylated in the bacterial cytosol to yield the active CyaA toxin, able to intoxicate and impair the physiology of immune cells. However, the relationships between post-translational modification and the folding and cytotoxic activities of CyaA remain elusive. Here, using a combination of biophysical approaches, including SEC-SAXS, HDX-MS and SR-CD, we show that calcium-induced disorder-to-order transitions and acylation are involved in CyaA secretion and folding into a compact and functional state. Our data sheds light on the complex relationship between post-translational modifications, structural disorder and protein folding. We propose a refolding model that is dependent on calcium and driven by local and distal acylation-dependent interactions within CyaA. Coupling calcium-binding and acylation-driven folding is likely also pertinent for other toxins produced by many Gram-negative bacterial pathogens.

**Keywords:** acylation; adenylate cyclase; folding

### 4.4. Role of Duplication in Evolution of Venoms

Ivan Koludarov ^1,2,^*, Timothy N. W. Jackson ^3^ and Bjoern Von-Reumont ^1,2^

^1^  Institute for Insect Biotechnology, Justus Liebig University, 35394 Giessen, Germany
^2^  Animal Venomics group, Fraunhofer Institute for Molecular Biology and Applied Ecology, 35394 Giessen, Germany
^3^  Australian Venom Research Unit, University of Melbourne, 3052 Melbourne, Australia

* Correspondence: Ivan.Koludarov@agrar.uni-giessen.de

**Abstract:** All venom proteins evolved from physiological proteins; however, the mechanisms of that transition are not known. The canonical way of thinking about this matter relies heavily on gene duplication to provide the relaxed selection constraints that are deemed necessary for the gene to acquire a new function. However, several recent studies have challenged that paradigm, providing examples of single gene recruitment. In our research, we looked into some of the major toxin families of reptiles and discovered that all of them evolve from families with widespread copy number variation. However, the transition from physiological gene to venom gene seems to happen via change of function followed by duplication, not the other way around as is commonly assumed. It is unlikely that all venom genes in all the lineages of venomous animals have evolved that way, but it is abundantly clear that the currently established paradigms cannot satisfyingly fit these results, necessitating the creation of more sophisticated models of gene evolution, as well as prompting new research agendas to validate them.

**Keywords:** evolution of venom genes; gene duplication; gene neofunctionalisation

### 4.5. Diversity in the Binding Interactions of Nicotinic Ligands and Toxins to the nAChRs and Associated Conformational Fluctuations-Insights into the Core Motif Dictating Antagonism

Yves Bourne ^1^, Gerlind Sulzenbacher ^1^, Zoran Radić ^2^, Laurent Chabaud ^3^, Rómulo Aráoz ^4^, Evelyne Benoit ^4^, Armen Zakarian ^5^, Denis Servent ^4^, Catherine Guillou ^3^, Palmer Taylor ^2^, Jordi Molgó ^4^ and Pascale Marchot^1,^*

^1^  ‘Architecture et Fonction des Macromolécules Biologiques’ laboratory, CNRS/AMU, Faculté des Sciences-Campus Luminy, 13288 Marseille CEDEX 09, France
^2^  Skaggs School of Pharmacy and Pharmaceutical Sciences, Dept. Pharmacology, University of California San Diego, La Jolla, CA 92093, USA
^3^  Institut de Chimie des Substances Naturelles, CNRS, 91198 Gif-sur-Yvette, France
^4^  Institut des Sciences du Vivant Frédéric Joliot, CEA Paris-Saclay, 91191 Gif-sur-Yvette, France
^5^  Department of Chemistry and Biochemistry, University of California Santa Barbara, Santa Barbara, CA 93106, USA

* Correspondence: pascale.marchot@univ-amu.fr

**Abstract:** The pentameric ACh-binding proteins (AChBP) from water snails are soluble structural and pharmacological surrogates of the extracellular, ligand-binding domain of nicotinic ACh receptors (nAChRs) [1]. As such, they offer pertinent models for studying the modes of binding of nicotinic effectors and the associated conformational changes, and correlate them with the functional alteration of the nAChR channel. The crystal structures of AChBP complexes revealed that nicotinic agonists and competitive antagonists bind primarily within a nest of aromatic side chains, contributed by loops C and F, which are located on opposing faces of each subunit interface, and induce a range of loop C conformations that modulate the size and shape of the binding pocket [2–4]. The macrocyclic imine phycotoxins belong to an emerging class of chemical agents associated with marine algal blooms and shellfish toxicity. Binding and voltage-clamp recordings on muscle-type and neuronal nAChRs revealed subnanomolar affinities dictated by slow dissociation, potent antagonism, and varying levels of nAChR subtype selectivity. The crystal structures of the complexes showed that common AChBP determinants imbedded within the aromatic nest confer high-affinity binding to the toxins, while the distinctive determinants brought about by loop F and located within the nest, or extending outside the nest towards apical, radial or ‘membrane’ subsites of the interface, dictate either broad or narrow nAChR subtype selectivity by the toxins [5−7]. Based on these data, new organic compounds aimed at pinpointing the minimal chemical motif that dictates antagonism were designed, synthetized and analyzed relative to nAChRs and AChBP [8]. Structural analysis of the complexes showed that the spiroimine core common to these compounds is the major component of their mode of binding, while the surrounding substituents are involved in nAChR subtype specificity. These data identify distinctive ligands, functional determinants and binding sites for the design of new drugs targeting disease-associated nAChR subtypes.

**Keywords:** AChBP; antagonism; macrocylic imine toxin; nicotinic ACh receptor; structure-function relationships

**Funding:** Works supported by grants from the CNRS-DREI (YB, PM), FRISBI (AFMB lab), NIH/NIGMS (AZ), USPHS (PT), ANR (JM).

**References**

1. Smit, A.B.; Brejc, K.; Syed, N.; Sixma, T.K. Structure and function of AChBP, homologue of the ligand-binding domain of the nicotinic acetylcholine receptor. *Ann. N. Y. Acad. Sci.* **2003**, *998*, 81–92.
2. Bourne, Y.; Talley, T.T.; Hansen, S.B.; Taylor, P.; Marchot, P. Crystal structure of a Cbtx–AChBP complex reveals essential interactions between snake α-neurotoxins and nicotinic receptors. *EMBO J.* **2005**, *24*, 1512–1522.
3. Hansen, S.B.; Sulzenbacher, G.; Huxford, T.; Marchot, P.; Taylor, P.; Bourne, Y. Structures of Aplysia AChBP complexes with nicotinic agonists and antagonists reveal distinctive binding interfaces and conformations. *EMBO J.* **2005**, *24*, 3635–3646.
4. Hibbs, R.E.; Sulzenbacher, G.; Shi, J.; Talley, T.T.; Conrod, S.; Kem, W.R.; Taylor, P.; Marchot, P.; Bourne, Y. Structural determinants for interaction of partial agonists with acetylcholine binding protein and neuronal α7 nicotinic acetylcholine receptor. *EMBO J.* **2009**, *28*, 3040–3051.
5. Bourne, Y.; Radić, Z.; Aráoz, R.; Talley, T.T.; Benoit, E.; Servent, D.; Taylor, P.; Molgó, J.; Marchot, P. Structural determinants in phycotoxins and AChBP conferring high affinity binding and nicotinic AChR antagonism. *Proc. Natl. Acad. Sci. USA* **2010**, *107*, 6076–6081.
6. Bourne, Y.; Sulzenbacher, G.; Radić, Z.; Aráoz, R.; Reynaud, M.; Benoit, E.; Zakarian, A.; Servent, D.; Molgó, J; Taylor, P.; et al. Marine macrocyclic imines, pinnatoxins A and G: Structural determinants and functional properties to distinguish neuronal α7 from muscle α12βγδ nAChRs. *Structure* **2015**, *23*, 1106–1115.
7. Molgó, J.; Marchot, P.; Aráoz, R.; Benoit, E.; Iorga, B.I.; Zakarian, A.; Taylor, P.; Bourne, Y.; Servent, D. Cyclic imine toxins from dinoflagellates: A growing family of potent antagonists of the nicotinic acetylcholine receptors. *J. Neurochem.* **2017**, *142*, 41–51.
8. Marchot & coll, manuscript in preparation.

### 4.6. A Clostridial-Like Neurotoxin That Selectively Targets *Anopheles* Mosquitoes

Geoffrey Masuyer ^1,2,^*, Estefania Contreras ^3^, Nadia Qureshi ^3^, Swati Chawla ^3^, Harpal S. Dhillon ^3^, Ham Lim Lee ^4^, Jianwu Chen ^3^, Pål Stenmark ^2,5^ and Sarjeet S. Gill ^3^

^1^  Department of Pharmacy and Pharmacology, University of Bath, Bath BA2 7AY, UK
^2^  Department of Biochemistry and Biophysics, Stockholm University, 10691 Stockholm, Sweden
^3^  Department of Molecular, Cell and Systems Biology, University of California Riverside, Riverside, CA 92521, USA
^4^  Unit of Medical Entomology, Institute for Medical Research, Kuala Lumpur 50588, Malaysia
^5^  Department of Experimental Medical Science, Lund University, 22100 Lund, Sweden

* Correspondence: gm283@bath.ac.uk


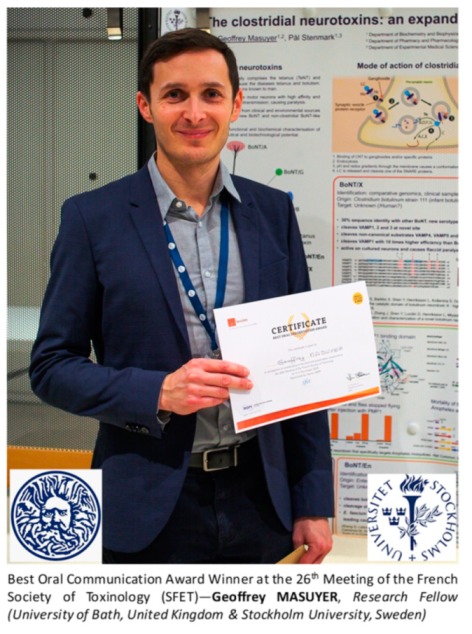


**Abstract:** Clostridial neurotoxins are potent toxins that target the nervous system of vertebrates causing paralytic diseases such as tetanus and botulism. Here, we present a new member of this family of toxins: PMP1, a clostridial-like neurotoxin that selectively targets *Anopheles* mosquitoes, thus expanding the range of host species targeted by this family. PMP1 [1] was isolated from *Paraclostridium bifermentans* strains collected in geographically varied, anopheline endemic areas. PMP1 was shown to use a common mechanism of toxicity that disrupts SNARE-mediated exocytosis by the cleavage of syntaxin. Our results suggest that PMP1 employs a different receptor recognition strategy, illustrated by the high-resolution structure of the PMP1-binding domain. The discovery of PMP1 has a significant impact on our understanding of clostridial neurotoxins’ evolution. Importantly, it provides an exciting opportunity for the development of innovative biotechnological tools that can reduce malaria through anopheline control.

**Keywords:** botulinum; *Clostridium*-like neurotoxin; mosquitocidal; tetanus

**Reference**

1. Contreras, E.; Masuyer, G.; Qureshi, N.; Chawla, S.; Dhillon, H.S.; Lee, H.L.; Chen, J.; Stenmark, P.; Gill, S.S. A neurotoxin that specifically targets *Anopheles* mosquitoes. *Nat. Commun.* **2019**, *10*, 2869.

### 4.7. The Neurotoxin Veratridine Induces Vasorelaxation of Murine Mesenteric Arteries, Unmasking a Cross-Talk Between Nav Channels, NCX Exchanger and eNO-Synthase

Joohee Park, César Mattei, Coralyne Proux, Daniel Henrion, Claire Legendre and Christian Legros *

MitoVasc Laboratory, Team 2 “CARdiovascular MEchanotransduction” UMR CNRS 6015–Inserm U1083, University of Angers, 49100 Angers, France

* Correspondence: christian.legros@univ-angers.fr

**Abstract:** Blood vessel cells express voltage-gated Na^+^ channels (Nav channels) and their activation induces a Ca^2+^ response mediated by Na^+^/Ca^2+^ exchangers (NCX) in Ca^2+^ entry mode. Nevertheless, the physiological role of Nav channels in vascular tissue is still controversial. The aim of our study was to identify the Nav channel subtypes in resistance artery and to define their contribution to the regulation of their vasomotricity by physiological and pharmacological approaches. To this end, we used mesenteric arteries (MA), as a suitable model of resistance artery from 5-month-old mice (C57Bl6/J, male and female). Our RT-qPCR data showed the expression of three transcripts encoding Nav1.2 (scn2a), Nav1.3 (scn3a) and Nav1.5 (scn5a) in MA. Presence of Nav channels in these arteries was confirmed by histoimmunostaining. Surprisingly, the activation of Nav channel by veratridine (VTD) induced the vasorelaxation of MA, monitored by wire myography. This VTD-induced vasorelaxation was totally abolished by tetrodotoxine (300 µM), L-NNA (a NO synthase inhibitor), indicating that the activation of TTX-sensitive Nav channels mediates the stimulation of the eNO-synthase (eNOS). Next, we investigated NCX’s implication in this pathway. We established the gene expression profile of NCX in murine MA by RT-qPCR, revealing the detection slc8a1 and slc8a2, encoding NCX1 and NCX2. In presence of the NCX inhibitor, KB-R7943, the relaxation induced by VTD was almost abolished. Altogether, our data highlight for the first time the role of Nav channels in the vasorelaxation response in murine MA. The activation of Nav channels induces Na^+^ entry and the subsequent membrane depolarization, which both trigger Ca^2+^ entry through NCX. This possible Nav channels–NCX–NOS cross-talk reflects the link between Na^+^ and Ca^2+^ homeostasis in vascular cells.

**Keywords:** mesenteric artery; NCX exchanger; vascular function; veratridine; voltage-gated Na^+^ channel

### 4.8. Cryptophycin–How the Culture Conditions Affect the Production of a Toxin with Anticancer Potency from Cyanobacteria

Alexandros Polyzois * and Sylvie Michel

Laboratoire des Produits naturels, Analyse et Synthèse, CITCOM-Cibles Thérapeutiques et Conception de Médicaments, UMR 8038 CNRS-Faculté de Pharmacie de Paris, Université Paris Descartes, 4 avenue de l’Observatoire, 75006 Paris, France

* Correspondence: polyzois@etu.parisdescartes.fr

**Abstract:** Cryptophycin-1 is a toxin naturally produced by filamentous cyanobacteria as a secondary metabolite. Its toxicity is due to tubulin inhibitor effects and to the cell cycle stop in the G2/M phase. Thus, it was examined as novel anticancer agent and it reached clinical phase II in 2002. However, the total synthesis of cryptophycin faces stereoselectivity issues and its yield is only moderate (<13%), and unsuitable for industrial production^1^. These considerations provide a rationale to investigate alternative solutions, like stressing the environmental conditions of the culture (ATCC53789) in order to find the optimal condition for metabolite over-production (more than 0.52 mg/L of culture)^2^. Here, we examine the effect of light photoperiod, light wavelength, light intensity and media composition. To examine the effect of light photoperiod, we tested constant and partial light (24:0, 16:8 Light:Dark), while, for light wavelength, color films were applied to test the effect of red and blue light. Low, medium and high light intensity experiments were performed in order to test the effect of photoinhibition. To look at medium composition, the nitrogen concentration was examined to test the implication of heterocysts, a N-fixing cell type. The culture was cultivated in an incubator at 25 °C and 120 rpm. For a 10 day period, the biomass of a 20 mL sample was extracted every second day, and then metabolite concentration was calculated through HPLC. Lastly, the “growth/time” and “metabolite concentration/time” curves were drawn. Remarkably, photoperiod has an opposite effect on the two curves. Regarding growth, the optimal period was 16:8 > 24:0, however, the inverse occurred for cryptophycin production. It is shown that even if the presence of non-light is needed for cell growth, metabolite production is higher under constant light conditions. Regarding the nitrogen concentration, and therefore the implication of heterocysts, it was noticed that they were not involved in the depsipeptide expression process. Moreover, the differentiation of light wavelength has a major effect on the culture, as the red filter contributed to a noticeable increase in culture growth. At last, the microorganism prefers medium light intensities for both growth and metabolite expression, as 80 μE was the optimal. These stress-test results contribute to the formation of the optimal culture conditions for metabolite overproduction, which would further the research and development of a novel potential anticancer drug.

**Keywords:** anti-cancer; cryptophycin; cyanobacteria

**References**

1. Eissler, S.; Stoncius, A.; Nahrwold, M.; Sewald, N. The synthesis of cryptophycins. *Synthesis* **2006**, *22*, 3747–3789.
2. Back, S.; Liang, J. Production of cryptophycin from blue-green algae. *J. Young Investig.* **2005**, *12*.

### 4.9. Alpha Neurotoxin Resistance among Vertebrates

Muzaffar Khan ^1^, Daniel Dashevsky ^2^, Dursan Kordis ^3^, Bryan Fry ^2^ and Michael Richardson^1,^*

^1^  Institute of Biology, Leiden University (IBL), Sylvius Laboratory (room 6.5.14b), Sylviusweg 72, 2333 Leiden, The Netherlands
^2^  School of Biological Sciences, The University of Queensland, St. Lucia, QLD 4072, Australia
^3^  Jožef Stefan Institute, Department of Molecular and Biomedical Science, University of Slovenia, 1000 Ljubljana, Slovenia

* Correspondence: m.k.richardson@biology.leidenuniv.nl

**Abstract:** A few animals have evolved molecular resistance to alpha-neurotoxins, most famously the mongoose and cobra. The resistance consists of one or two base-pair changes in the ligand-binding of the nicotinic acetylcholine receptor (nAChR) with possible posttranslational modification of the receptor protein. This leads to reduced binding of snake alpha neurotoxins to the receptor without a major effect on the physiological binding of acetylcholine. We have sequenced the ligand-binding domain of the acetylcholine in a wide range of vertebrate taxa and looked for evidence of resistance-related changes in the coding sequence. We find that several snake lineages and lizards, but not birds, have evolved such changes. The lack of resistance-related changes in birds is surprising, given the prevalence of ophiophagy in the species examined. Functional studies in embryos confirmed that the chicken is six times more susceptible to cobra venom than *Pogona vitticeps*, which is putatively resistant, based on our sequencing of its nAChR. Bioinformatics analysis showed evidence that putative resistance-related sites in the nAChR are under positive selection.

**Keywords:** alpha-neurotoxin; nicotinic acetylcholine receptor; resistance-related site; vertebrate

### 4.10. Maurocalcin Analogue MCaE12A Alters Calcium Homeostasis in Cardiomyocytes Via Regulation of RyR2 Activity

Stephan De Waard ^1^, Jérôme Montnach ^1^, Charly Cortinovis ^1^, Olfa Chkir ^1^, Morteza Erfanian ^1^, Nathalie Gaborit ^1^, Patricia Lemarchand ^1^, Pietro Mesirca ^2,3^, Isabelle Bidaud ^2,3^ Matteo E. Mangoni ^2,3^, Michel De Waard ^1,3^ and Michel Ronjat^1,3,^*

^1^  Institut du thorax, Inserm UMR 1087/CNRS UMR 6291, University of Nantes, 44007 Nantes, France
^2^  IGF CNRS UMR 5203, Inserm U1191 University of Montpellier, 34094 Montpellier, France
^3^  LabEx “Ion Channels, Science & Therapeutics”, Florian Lesage, Institut de Pharmacologie Moléculaire et Cellulaire, Université Côte d’Azur, CNRS, Sophia Antipolis, 06550 Valbonne, France

* Correspondence: michel.ronjat@univ-nantes.fr

**Abstract:** The ryanodine receptor type 2 (RyR2) is the Ca^2+^ channel that controls cardiomyocytes contraction in response to an inward Ca^2+^ current induced by the action potential. RyR2 gating behaviour is strictly controlled by cytoplasmic Ca^2+^ concentration and by a number of proteins, whose functions are themselves controlled by Ca^2+^. RyR2 represents an important target for treatment of cardiac pathologies resulting from, or resulting in, a defect in the cardiomyocytes Ca^2+^ homeostasis. Maurocalcine (MCa), a 33 amino acids toxin, originally isolated from scorpion venom, has been previously characterized as a high-affinity activator of the skeletal isoform of RyRs. We thus investigated the effects of the MCa analogue MCaE12A on (1) the sensitivity of isolated RyR2 to Ca^2+^, (2) depolarization-induced Ca^2+^ movements in adult rat cardiomyocytes and on human iPSC-derived cardiomyocytes. We demonstrate that MCaE12A is a high-affinity modulator of RyR2 and therefore constitutes an important tool for studying RyR2 structure-to-function, as well as manipulating Ca^2+^ homeostasis and dynamics in cardiac cells.

**Keywords:** heart; ryanodine receptor; scorpion toxin

### 4.11. Recombinant Claudin Molecules as Innovative Capture Structures for *Clostridium perfringens* Enterotoxin

Jasmin Weisemann ^1^, Stefan Mahrhold ^1^, Maren Kruger ^2^, Thea Neumann ^2^, Brigitte G. Dorner ^2^ and Andreas Rummel^1,^*

^1^  Medizinische Hochschule Hannover, Institut für Toxikologie, 30625 Hannover, Germany
^2^  Robert Koch Institut, Biological Toxins (ZBS 3), Centre for Biological Threats and Special Pathogens, 13353 Berlin, Germany

* Correspondence: rummel.andreas@mh-hannover.de

**Abstract:***Clostridium perfringens* causes a broad spectrum of diseases in animals and humans. Depending on the production of four major toxins, it is classified into five toxinotypes, A–E. The most important of the 12 minor toxins produced is the *C. perfringens* enterotoxin (CPE), the second largest cause of most cases of bacterial food-borne illnesses and antibiotic-associated diarrhea. This makes CPE an important analyte in clinical differential diagnostics. In contrast, CPE is also explored as a potential anticancer agent. CPE is a 35 kDa β, pore-forming toxin classified into the aerolysin family, which oligomerizes upon receptor recognition and subsequently forms cation-selective pores in the membrane of epithelial cells, thereby inducing cell death. Here, we aim at designing a capture structure specific for functionally active CPE, which will be implemented into a rapid detection system. The C-terminal 15 kDa domain of the CPE constitutes the receptor-binding domain (CPE-RBD), which recognizes claudins (CLDN), a family of 20-27 kDa tetraspanin proteins forming tight junctions between epithelial cells. Robust binding of CPE to CLDN-3 and-4 and weaker binding to CLDN-1,-6,-7,-8,-9,-14 and-19 has been reported. We explored CLDN-1,-3,-4 and-19 as CPE capture structure candidates. Various fusion proteins and truncation mutants of CLDNs were recombinantly expressed in eukaryotic cell lines and E. coli. The isolated 15 kDa CPE-RBD served as prey or bait in various pull-down assays and co-immunoprecipitations with CLDN mutants. We identified CLDN-4 among CLDN-1,-3,-4 and-19 as the best binder and, in contrast to previous reports, determined full-length CLDN-4 as being essential for high-affinity binding to CPE-RBD, as well as full-length recombinant CPE. Various CLDN-4 constructs were explored and tested for expression and isolation to obtain a soluble, pure and functionally folded CLDN-4. The binding kinetics of CPE-RBD to CLDN mutants were determined by SPR experiments. The optimization of detergent allowed its spotting on a gold-chip to serve in a rapid detection system. The optimal CLDN-4 capture structure, comprising four transmembrane domains, was isolated in acceptable yield and good purity and displayed sub-nanomolar binding affinity constants towards CPE. Currently, this structure is being implemented into a rapid detection system.

**Keywords:** claudin; *Clostridium perfringens*; enterotoxin

### 4.12. Bio-Guided Fractionation for the Isolation of Antiplasmodial and Cytotoxic Compounds from the Venom of *Bufo* Toads

Mathilde Wells ^1,^*, Laura Soumoy ^2^, Fabrice Journé ^2^, Sven Saussez ^2^, Stéphanie Hambye ^1^ and Bertrand Blankert ^1^

^1^  Laboratory of Pharmaceutical Analysis, Faculty of Medicine and Pharmacy, Research Institute for Health Sciences and Technology, University of Mons, Bât. Mendeleiev, 7000 Mons, Belgium
^2^  Laboratory of Human Anatomy and Experimental Oncology, Faculty of Medicine and Pharmacy, Research Institute for Health Sciences and Technology, University of Mons, Bât. Pentagone, 7000 Mons, Belgium

* Correspondence: mathilde.wells@umons.ac.be

**Abstract:** Malaria remains a major concern for health organizations around the world. In 2017, the World Health Organization reported more than 219 million cases and 435,000 deaths. With 87 countries affected, more than 800 million people are at risk of infection. The emergence and transmission of resistances to most antimalarial drugs is a real worry. Thus, the need for new therapeutic candidates is an absolute necessity [1]. In recent years, animal venoms and secretions have sparked a growing interest in scientists. In fact, toad venoms constitute a rich source of molecules, mainly bufadienolides, with many potential therapeutic activities [2]. The objective of this on-going project is to develop a bio-guided fractionation process and the subsequent discovery of new drug candidates against malaria from toad venom. Raw extract characterization: Multiple *Bufo* species will be considered during this work. Up to now, two species have been studied: *Rhinella marina* and *Bufo bufo*. The extraction process from the air-dried gland secretions of the *Bufo* toads consists of an ultrasonication-assisted solvent extraction. Two solvents have been tested: methanol and acetonitrile. The venom composition is subject to variability between batches depending on the animal’s habitat and its diet. After each extraction, the raw extracts are analyzed by TLC and LC-MS to provide an overview of the compounds present in the sample. Fractionation process: during this step, flash chromatography is considered as the first approach, to obtain rough fractions that will also be analyzed by TLC and LC-MS and then biologically studied. Flash chromatography offers a fast and simple separation process that can be applied to complex natural products. In the first fractionation round, three to four fractions are obtained. The following step will consist of producing subfractions of the fractions displaying interesting therapeutic properties. For this purpose, further preparative techniques will be considered such as flash chromatography and semi-preparative HPLC. Biological activity: each raw extract and the subsequently obtained fractions are tested for their antiplasmodial activity (3D7 and W2 strains) using the pLDH assay and microscopic evaluation. Their cytotoxicities are also assessed on a panel of human cell lines. A parallel project aims to evaluate the effect of the above-mentioned extracts and fractions on several human melanoma cell lines that have developed a resistance to targeted therapies. The samples that display antiplasmodial activities and/or cytotoxic activities against melanoma cells will be further analyzed (LC-MS) and structurally characterized by NMR analysis (^1^H-NMR, ^13^C-NMR, COSY).

**Keywords:** antiplasmodial; toad; venom

**References**

1. *WHO World Malaria Report*; WHO: Geneva, Switzerland, 2018.
2. Rodriguez, C.; Rollins-Smith, L.; Ibanez, R.; Durant-Archibold, A.A.; Gutierrez, M. Toxins and pharmacologically active compounds from species of the family Bufonidae (Amphibia, Anura). *J. Ethnopharmacol.* **2017**, *198*, 235–254.

## 5. Poster Presentations (When More Than One Author, the Underlined Name Is That of the Presenter)

### 5.1. Relationship between Cardiac Oxidative Stress Pathways and Circadian Rhythms after Scorpion Envenomation

Fares Daachi, Sonia ADI-Bessalem *, Amal Megdad-lamraoui and Fatima Laraba-Djebari

USTHB, Faculty of Biological Sciences, Laboratory of Cellular and Molecular Biology, BP 32 El-Alia, Bab Ezzouar, 16111 Algiers, Algeria

* Correspondence: soniabessalem@hotmail.com

**Abstract:** Scorpion venom is known to cause the liberation of neurotransmitters and the release of several inflammatory mediators, like cytokines, eicosanoids, nitric oxide and reactive oxygen species (ROS). These latter are important contributors to heart failure and disease. However, little is known about how circadian rhythms, or rhythm desynchrony, are involved in these key pathologic stress responses. The aim of this study is to investigate the role of the circadian rhythm on the cardiac oxidative stress pathways, and indicate how free radical biology coincides with the pathogenesis of the cardiovascular system. Twelve NMRI mice were envenomed with a sublethal dose of *Androctonus australis hector* venom (Aah) (0.75 mg/kg, s.c) during light (at 1 HALO, n = 6) and dark phases (at 18 HALO, n = 6) in order to investigate the circadian variations in pro-oxidant parameters, antioxidant defenses and lipid peroxidation. Evaluation of the myeloperoxidase activity as a quantitative assessment of neutrophil infiltration, vascular permeability, as well as a histopathological analysis of cardiac tissue, was also performed in the two groups (1 HALO and 18 HALO). Higher levels of nitrite (*p* < 0.0001), hydrogen peroxide (*p* < 0.0001) and lipid peroxidation (*p* < 0.0001) were detected in evening-excised hearts, associated with a lower (*p* < 0.05) myeloperoxidase activity. For the antioxidant defenses, the catalase activity increased during the light phase, while depletion in GSH concentration was observed at the dark phase. Moreover, a greater extravasation of Evans blue (*p* < 0.01) was detected in the myocardial homogenates of the dark phase group, as compared to light phase hearts. The histopathological alterations were similar in the two phases. In conclusion, a higher oxidative stress seems to be operative in the mouse heart during the middle of the dark phase. An imbalance of antioxidant defences, and/or a higher radical generation and unsaturation degree of bio-membranes lipids, may be hypothesized to favour myocardial oxidative stress at the motor activity phase in mice. This is an entirely new frontier of investigation, leading to new understanding and avenues for treating heart disease.

**Keywords:** antioxidant; cardiac oxidative stress; circadian rhythm; scorpion venom

### 5.2. Potential New Treatment for Scorpion Envenomation Pathogenesis: Avian Antibodies (IgYs) Associated to Histamine H4-Receptor Antagonist

Amal Megdad-Lamraoui, Sonia Adi-Bessalem *, Amina Sifi and Fatima Laraba-Djebari

USTHB, Faculty of Biological Sciences, Laboratory of Cellular and Molecular Biology, BP 32 El-Alia, Bab Ezzouar, 16111 Algiers, Algeria

* Correspondence: soniabessalem@hotmail.com

**Abstract:** Organ dysfunction during scorpion envenomation could be attributed to the activation of a complex inflammatory process, characterized by several inflammatory mediators releasing vasoactive mediators, such as histamine. Immunotherapy constitutes the specific treatment although different approaches have been developed to treat the deleterious effects of the venom. Nevertheless, the use of mammalian antivenoms, which are F(ab’)2 immunoglobulin fragments purified from the blood of hyperimmunized horses with scorpion venom, may cause adverse effects due to the host’s immune system activation. In addition to immunotherapy, symptomatic treatment is currently administered (analgesics, antipyretics, antihypertensives, anticonvulsants and steroids). The aim of the current study is to develop an appropriate therapy for severe envenomation cases. We evaluate the effects of egg yolk antibodies (IgYs) purified from hyperimmunized chicken with *Androctonus australis hector* (Aah) scorpion venom, alone or associated with a histamine H4-receptor antagonist (JNJ-7777120), against the pulmonary and splenic inflammatory response and tissue alteration induced by Aah scorpion venom. The egg yolk antibodies and the histamine H4-receptor antagonist were administered thirty minutes after the experimental envenomation. The inflammation response was evaluated 24 h after venom injection by the estimation of vascular permeability changes, infiltration of inflammatory cells, oxidative stress markers, and histological analysis, as well as metabolic enzyme release in mice sera. The results showed that scorpion venom induced inflammatory disorders characterized by an increase in inflammatory cell infiltration and levels of reactive oxygen/nitrogen species, lipid peroxidation, and a decreased antioxidant defense. Moreover, significant alterations in the pulmonary and the splenic tissues were also observed. The administration of the IgYs antibodies fragments to mice after venom inoculation resulted in a decrease of leukocyte infiltration as well as a decrease in the vascular permeability amount. A marked reduction in oxygen species levels, membrane lipids peroxidation, and an increase in antioxidant levels with decreased pulmonary and splenic tissue alteration, were also observed. The association of IgYs fragments and the histamine H4-receptor antagonist resulted in more significant reduction of inflammatory and oxidative stress markers. In addition, a reduction in the perturbation of the lung and spleen tissue structure and metabolic enzyme levels was observed after the addition of these two treatments. These results indicate that the immunotherapy with histamine H4-receptor antagonist exhibits potent therapeutic effects against scorpion venom-induced inflammation response and oxidative/nitrosative stress in pulmonary and splenic tissues and offers the possibility of the use of IgYs antibodies associated to the histamine H4-receptor antagonist in the treatment of scorpion venom-induced immune-inflammatory disorders.

**Keywords:** chicken egg yolk antibody (IgYs); histamine H4 receptor; inflammatory response; immunotherapy; scorpion envenomation

### 5.3. ALERTOX-NET: Atlantic Area Network for Innovative Toxicity Alert Systems for Safer Seafood Products: Towards Fast Early Warning Detection Systems for Marine Toxins

Rómulo Aráoz ^1,2,^*, Fanny Noirmain ^2^, Meena Murmu ^2^, Jordi Molgó ^2^ and Denis Servent ^2^

^1^  CNRS, Institut de Neurosciences–Paris Saclay UMR9197, Université Paris-Saclay, 91198 Gif-sur-Yvette, France
^2^  Service d’Ingénierie Moléculaire des Protéines CEA/DRF/JOLIOT/SIMOPRO, Université Paris-Saclay, 91191 Gif-sur-Yvette, France

* Correspondence: romulo.araoz@cea.fr

**Abstract:** The ALERTOX-NET project is funded by the INTERREG Atlantic Area European Regional Development fund. The project aims to develop an easy-to-use detection and alert system for emerging marine toxins. The project will utilize state-of-the-art toxicity detection systems and disseminate results to all end users. Under the coordination of Prof. Luis Botana, partners from eleven centers of excellence in Spain (four centers), Portugal (one), France (one), the United Kingdom (two) and Ireland (two) bring together a wealth of experience to provide innovative solutions to achieve the project deliverables. ALERTOX-NET is an inter-laboratory collaborative effort for the development and integration of an alert system for marine toxins, considering environmental factors and linking the Seafood Industries’ needs and consumers’ health protection. ALERTOX-NET will grow into a cluster of excellence in seafood toxicity issues by (a) contributing data and technological innovation; (b) involving the main Regulatory Agencies in adapting the legislation to the phenomena of emerging toxins and advising/informing about new detection systems. The ALERTOX-NET objectives are to a) identify industrial needs regarding marine toxins detection; b) develop innovative toxicological alert systems for more safety seafood; (c) develop easy-to-use toxicity alert systems for the industrial sector; (d) develop the information exchange network ALERTOX-NET at the EU level. In the frame of ALERTOX-NET, we participated in the *Capitalization* workpackage (WP), by creating a Regional Working Group in close interaction with IFREMER French partner, inviting stakeholders to joint our network and discussing biotech views and needs with them regarding marine toxin detection. We also participated in the *Identification industry needs and detection methods* WP, by editing a Catalogue of Methods and Procedures focused on Emergent Marine Toxins in Europe (tetrodotoxins, palytoxins and cyclic imine toxins) in close interaction with all partners. Further, we characterized the antagonistic activity of prorocentrolide and portimine on muscle-type and neuronal nicotinic acetylcholine receptors, thereby increasing knowledge of this large family of cyclic imine toxins. Next, we initiated a novel approach for studying the impact of cyclic imine toxins on zebrafish larvae. In the *Joint Design of an Easy-to-Use Toxicity Alert System* WP, we launched an inter-laboratory method comparison to test the performance of our developed methods, namely the microplate-receptor binding assay (WO2012101378 A1) and the lateral flow test NeuroTorp (WO2017108115 A1), to detect cyclic imine toxins. Both methods are based on a new concept for these technologies: the high affinity of the toxins for their receptor targets. In the frame of the *Industrial Pilot of the Toxicity Alert System* WP, we contacted several stakeholders to inquire about their needs in the field of marine toxin detection and proposed NeuroTorp for industrial piloting. Taking advantage of the fact that NeuroTorp is a fast and cost-effective early warning device for in-field detection of marine neurotoxins by end-users Novakits, a Biotech located at Nantes, France, will pilot the performance of this lateral flow test in field conditions with the participation of shellfish farmers for the early detection of cyclic imine toxins. The final aim of ALERTOX-NET is to propose Guidelines for the integration/implementation of the Alert system for marine toxins.

**Keywords:** cyclic imine toxin; emergent marine toxin; HAB; method development

**Acknowledgments:** The authors acknowledge INTERREG Atlantic Area for funding the ALERTOX-NET EAPA_317/2016 project.

### 5.4. Study on the Affinity and Selectivity Improvement of the Spider Phlotoxin 1 for the Human 1.7 Subtype of Voltage-Gated Sodium Channels

Evelyne Benoit^1,2,^*, Tânia C. Goncalves ^1,3^, Pierre Lesport ^4^, Sarah Kuylle ^1^, Enrico Stura ^1^, Justyna Ciolek ^1^, Gilles Mourier ^1^, Denis Servent ^1^, Emmanuel Bourinet ^4^ and Nicolas Gilles ^1^

^1^  Service d’Ingénierie Moléculaire des Protéines (SIMOPRO), CEA, Université Paris-Saclay, 91191 Gif-sur-Yvette, France
^2^  Institut des Neurosciences Paris-Saclay (Neuro-PSI), UMR CNRS/Université Paris-Sud 9197, Université Paris-Saclay, 91198 Gif-sur-Yvette, France
^3^  Sanofi R&D, Integrated Drug Discovery–High Content Biology, 94440 Vitry-sur-Seine, France
^4^  Institut de Génomique Fonctionnelle (IGF), CNRS-UMR 5203, Inserm-U661, Université de Montpellier, Laboratories of Excellence-Ion Channel Science and Therapeutics, 34094 Montpellier, France

* Correspondence: evelyne.benoit@cea.fr

**Abstract:** Over the past two decades, venom toxins have been explored as alternatives to opioids to treat chronic debilitating pain. Approximately 20 potential analgesic toxins, mainly from spider venom, are known to inhibit the Nav1.7 subtype of voltage-gated sodium (Nav) channels with high affinity, making them the most promising genetically validated antinociceptive targets identified so far. The present study aimed to consolidate the development of phlotoxin 1 (PhlTx1), a 34-amino acid and 3-disulfide bridge peptide of a *Phlogiellus* genus spider, as an antinociceptive agent by improving its affinity and selectivity for the human (h) Nav1.7 subtype. The synthetic homologue of PhlTx1 was generated and, as the natural peptide, equilibrated between two active forms on reverse-phase liquid chromatography, and exhibited potent analgesic effects in a mouse model of inflammatory pain. The effects of PhlTx1 and eight successfully synthetized alanine-substituted variants were studied (by automated whole-cell patch-clamp electrophysiology) on cell lines stably overexpressing hNav subtypes, as well as two cardiac targets, the hCav1.2 and hKv11.1 subtypes of voltage-gated calcium (Cav) and potassium (Kv) channels, respectively. PhlTx1 and D7A-PhlTx1 were shown to inhibit hNav 1.1–1.3 and 1.5–1.7 subtypes at one hundred nanomolar concentrations, while their affinities for hNav1.4 and 1.8, hCav1.2 and hKv11.1 subtypes were over micromolar concentrations. Despite similar analgesic effects in the mouse model of inflammatory pain and selectivity profiles, the affinity of D7A-PhlTx1 for the Nav1.7 subtype was at least five times higher than that of the wild-type peptide. Computational modelling was performed to deduce the 3D-structure of PhlTx1 and to suggest the amino acids involved in the efficiency of the molecule. In conclusion, the present structure–activity relationship study of PhlTx1 results in a low improved affinity of the molecule for the Nav1.7 subtype, without any marked change in the molecule selectivity against the other studied ion channel subtypes. Further experiments are therefore necessary before considering the development of PhlTx1 or synthetic variants as antinociceptive drug candidates.

**Keywords:** human voltage-gated ion channel subtype; mouse model of Nav1.7-mediated pain; Nav1.7 channel subtype; *Phlogiellus* spider; phlotoxin 1

### 5.5. Immunopreventive Approach Based on Nanoformulation Using Encapsulated *Cerastes cerastes* Venom in Calcium-Alginate Nanoparticles

Asma Hamzoui and Fatima Laraba-Djebari *

USTHB, Faculty of Biological Sciences, Laboratory of Cellular and Molecular Biology, BP 32 El-Alia, Bab Ezzouar, 16111 Algiers, Algeria

* Correspondence: flaraba@hotmail.com

**Abstract:** Envenomation is a major health problem in many regions of the world with increasing prevalence in tropical regions. Snake envenomation is characterized by various effects, such as hemorrhage, inflammation, edema and necrosis. Snakes represent the most venomous animals; their venoms are a potential source of bioactive proteins, and each of them can cause serious disturbances. Immunotherapy is the only treatment; however, it has limited efficiency, due mainly to the delay in its administration. In this study, an immunopreventive approach based on vaccine nanoformulation, using *Cerastes cerastes* venom encapsulated in calcium–alginate nanoparticles, was designed in order to enhance the efficiency of the immune response. An immunization schedule was undertaken in mice by intranasal route to evaluate the potential of *C. cerastes* venom encapsulated in calcium–alginate nanoparticles against *C. cerastes* envenomation. The obtained results showed that this formulation stimulated the humoral immune response by inducing the production of high levels of specific IgG antibodies, conferring immunoprotection up to 6 LD_50_. This immune response was associated to a low systemic reactogenicity. The results also showed a moderate inflammatory response characterized by the recruitment of inflammatory blood cells, low myeloperoxidase (MPO) and eosinophil peroxidase (EPO) activities and a reduction in histopathological alterations. Calcium alginate nanoparticles appear to be a promising adjuvant system against envenomation by *C. cerastes* as they is able to improve the development of an effective humoral response and immunoprotection against the deleterious effects of severe envenomation.

**Keywords:** immunoprotection; nanoparticle; snake envenomation

### 5.6. Venom-Induced Hepato-Renal Damage: Role of Toll-Like Receptor 4 in Neutrophil-Mediated Inflammation, Nitrosative and Oxidative Stress

Dalila Khemili, Asma Kaddache, Fatima Laraba-Djebari and Djelila Hammoudi-Triki *

USTHB, Faculty of Biological Sciences, Laboratory of Cellular and Molecular Biology, University of Sciences and Technology Houari Boumediene, 16111 Algiers, Algeria

* Correspondence: hammoudid@gmail.com

**Abstract:** Systemic inflammatory response and the generation of oxidative stress contribute to scorpion venom-induced hepato-renal injury. Toll-like Receptors (TLRs), in particular TLR4, are a critical link between oxidative stress and inflammation. The TLR family of receptors are involved in alerting the immune system of microbial danger and the damage-associated molecular pattern molecules (DAMPs) that are released during oxidative stress conditions. These receptors are also implicated in the recognition of Venom-Associated Molecular Patterns (VAMPs). The present study was undertaken to investigate the involvement of TLR4 in venom-induced hepato-renal immunopathology, through the pharmacological targeting of TLR4 with the selective inhibitor TAK-242 (Resatorvid). The obtained results show that the systemic inhibition of TLR4 prevents hepato-renal neutrophil-mediated inflammation, induced by *Androctonus australis hector* venom, as revealed by a significant decrease in the neutrophil cell count in the peripheral blood, associated with a significant decline in neutrophils degranulation and sequestration to hepatic and renal tissues. Moreover, TAK-242 administration inhibited nitrite-level increases in serum, malondiadehyde (MDA) and protein carbonyls tissue content concomitantly with a significant increase in catalase activity and reduced glutathione (GSH) level in tissue homogenates. Furthermore, venom-induced increases in serum aminotransferases (ALT, AST), urea, and creatinine levels, which are indicative of hepato-renal damage, were significantly suppressed by pre-treatment with the TLR4 inhibitor, concordantly with a remarkable improvement in histological features. Our findings indicate that TLR4 signaling acts by modulating scorpion venom-induced hepato-renal inflammation, probably through the direct action of venom-associated molecular patterns (VAMPs) or indirectly through damage-associated molecular patterns (DAMPs) release.

**Keywords:***Androctonus australis hector* venom; hepato-renal injury; inflammation; oxidative stress; TLR4

### 5.7. Neuroinflammation and Demyelinating Murine Model: Effect of K^+^ Channel Blocker from Scorpion Venom

Hadjila Moussaoui ^1^, Amina Ladjel-Mendil ^1^, Marie-France Martin-Eauclaire ^2^ and Fatima Laraba-Djebari^1,^*

^1^  USTHB, Faculty of Biological Sciences, Laboratory of Cellular and Molecular Biology, BP 32 El-Alia, Bab Ezzouar, 16111 Algiers, Algeria
^2^  CRN2M CNRS/Aix Marseille Université UMR 7286, 13344 Marseille CEDEX 15, France

* Correspondence: flaraba@hotmail.com

**Abstract:** Scorpion toxins are powerful pharmacological tools to study the mechanisms of neurodegenerative diseases related to ionic channel dysfunction, such as the demyelination of the central nervous system (CNS). Several studies illustrate that kaliotoxin, a potent and highly selective blocker of the Kv1.1 and Kv1.3 potassium channels, can restore the conduction of demyelinated axons and potentiate synaptic transmission. In this study, the effect of kaliotoxin on the neuroinflammatory response was investigated in a murine demyelinating model induced by cuprizone. The obtained results showed that mice exposed to cuprizone over six weeks develop important neuro-immunological disorders characterized by severe alterations in the cerebral structure and function. These alterations were marked by a myelin degeneration, neuronal oedema and axonal loss associated with a neuro-immuno-inflammatory response. Furthermore, a low dose of kaliotoxin, injected by the intracerebroventricular route, seemed to reduce tissue alterations accompanied with a decrease in neuroinflammatory and oxidative stress markers. These data suggest that kaliotoxin is able to ameliorate neuronal conduction and reduce neuro-inflammation in the murine cuprizone-induced demyelinating model. Potassium channel blockers may represent useful therapeutic agents in demyelination-related diseases via the suppression of neuro-inflammation in the CNS.

**Keywords:** demyelinating model; K^+^ channel blocker; neuroinflammation

### 5.8. The Neurotoxin Veratridine Induces Vasorelaxation of Murine Mesenteric Arteries, Unmasking a Cross-Talk between Nav Channels, NCX Exchanger and eNO-Synthase

Joohee Park, César Mattei, Coralyne Proux, Daniel Henrion, Claire Legendre and Christian Legros *

MitoVasc Laboratory, Team 2 “CARdiovascular MEchanotransduction” UMR CNRS 6015–Inserm U1083, University of Angers, 49100 Angers, France

* Correspondence: christian.legros@univ-angers.fr

**Abstract:** Blood vessel cells express voltage-gated Na^+^ channels (Nav channels) and their activation induces a Ca^2+^ response mediated by Na^+^/Ca^2+^ exchangers (NCX) in Ca^2+^ entry mode. Nevertheless, the physiological role of Nav channels in vascular tissue is still controversial. The aim of our study was to identify the Nav channel subtypes in the resistance artery and to define their contribution to the regulation of their vasomotricity by physiological and pharmacological approaches. To this end, we used mesenteric arteries (MA), as a suitable model of resistance artery from 5-month-old mice (C57Bl6/J, male and female). Our RT-qPCR data showed the expression of three transcripts encoding Nav1.2 (scn2a), Nav1.3 (scn3a) and Nav1.5 (scn5a) in MA. The presence of Nav channels in these arteries was confirmed by histoimmunostaining. Surprisingly, the activation of the Nav channel by veratridine (VTD) induced the vasorelaxation of MA monitored by wire myography. This VTD-induced vasorelaxation was totally abolished by tetrodotoxine (300 µM), L-NNA (an NO synthase inhibitor), indicating that the activation of TTX-sensitive Nav channels mediates the stimulation of the eNO-synthase (eNOS). Next, we investigated the implication of NCX in this pathway. We established the gene expression profile of NCX in murine MA by RT-qPCR, revealing the presence of slc8a1 and slc8a2, encoding NCX1 and NCX2. In presence of the NCX inhibitor, KB-R7943, the relaxation induced by VTD was almost abolished. Altogether, our data highlight for the first time the role of Nav channels in vasorelaxation response in murine MA. The activation of Nav channels induces Na^+^ entry and subsequent membrane depolarization, which both trigger Ca^2+^ entry through NCX. This possible Nav channels–NCX–eNOS cross-talk reflects the link between Na^+^ and Ca^2+^ homeostasis in vascular cells.

**Keywords:** mesenteric artery; NCX exchanger; vascular function; veratridine; voltage-gated Na^+^ channel

### 5.9. High-Throughput Screening of Venom for Identification of Active Compound in Ion Channels

Ludivine Lopez^1,2,^*, Sébastien Nicolas ^1^, Lucie Jaquillard ^2^, Jérôme Montnach ^1^, Rémy Beroud ^2^ and Michel De Waard ^1^

^1^  Institut du thorax, Inserm UMR 1087/CNRS UMR 6291, LabEx “Ion Channels, Science & Therapeutics”, University of Nantes, 44007 Nantes, France
^2^  Smartox Biotechnology, 6 rue des Platanes, 38120 Saint Egrève, France

* Correspondence: ludivine.lopez@univ-nantes.fr


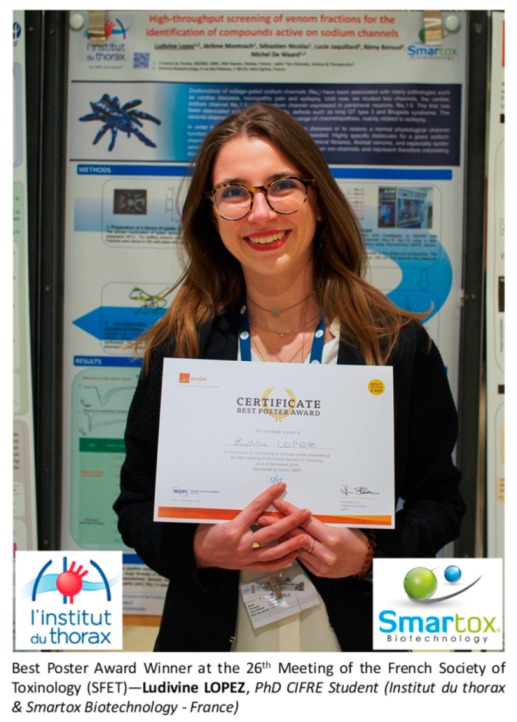


**Abstract:** Dysfunctions of voltage-gated sodium channels (Nav) have been associated with many pathological conditions such as cardiac diseases, neuropathic pain and epilepsy. In order to study the role of these channels in diseases or to restore function, specific molecules targeting ion channels are needed. Highly specific molecules for a given isoform of sodium channel are hard to discover with the usual chemical libraries. Animal venoms, and especially spider venom, contain tens of peptides acting on ion-channels and therefore represent interesting libraries for drug discovery. By screening spider venoms on Nav we aimed to identify new toxins specifically targeting one channel isoform, with the use of an automated patch-clamp (APC) technique (SyncroPatch364, Nanion). For that purpose, all venoms were preliminary fractionated in libraries of 64 fractions and tested on several stable Nav cell lines. APC allows one to test two venoms at the same time and accelerates the drug discovery process. Fractions of interest are those that reduce the sodium peak current (by at least 30%), slow-down inactivation, or increase the late sodium current. False-positive fractions were excluded based on detection of material in HPLC or mass spectrometry. Until now, two different Nav lines have been tested: Nav1.5 and Nav1.6. Primary screening allows for the identification of 28 fractions active in at least one isoform. Among them, 24 are specific for one channel (10 for Nav1.5, 14 for Nav1.6). The majority of positive fractions induce slow-down inactivation (five for Nav1.5, 12 for Nav1.6). The selected fractions are re-fractionated with a complementary purification technique until isolated peptides are obtained. These molecules are tested again for bioactivity until full de novo sequencing, chemical synthesis and full pharmacological characterization. This study suggests that, among the large number of toxins in venoms, a great variety targets sodium channels with specificity for each sodium channel isoform, and illustrates how ACP is essential for screening.

**Keywords:** automated patch-clamp; screening; sodium channel; spider toxin

### 5.10. Toxicity, Transfer and Depuration of Anatoxin-A (Neuroxin) in Medaka Fish Exposed by Gavage

Simon Colas and Benjamin Marie *

UMR 7245 CNRS/MNHN Molécules de Communications et Adaptations des Micro-Organismes, Sorbonne Universités, Muséum National d’Histoire naturelle, 75005 Paris, France

* Correspondence: bmarie@mnhn.fr

**Abstract:** The proliferation of cyanobacteria is increasingly prevalent in warm and nutrient-enriched waters and occurs in many rivers and water bodies, due especially to eutrophication. The aim of this work is to study the toxicity, the transfer and the depuration of the anatoxin-a, a neurotoxin produced by benthic cyanobacterial biofilms in female medaka fish. This work will provide answers regarding the acute toxicity induced by single gavage by anatoxin-a of medaka fish and the risks of exposure by the ingestion of contaminated fish fleshes, considering that data on these aspects remain particularly limited. The oral LD_50_ of a single dose of (±)-anatoxin-a was determined at 11.50 µg/g. First of all, a lethal dose (100% from 20 µg/g) provokes rapid respiratory paralysis (in 30–60 s) in the fish, inducing fish death by asphyxia. Noticeably, neither death nor an apparent compartmental effect occurred during the experimentation period for the 45 fish exposed to a single sub-acute dose of (±)-anatoxin-a corresponding to the non-observable effect level (NOEL = 6.67 µg/g). Subsequently, the toxicokinetics of the (±)-anatoxin-a was observed in the intestines, the livers and the muscles of female medaka fish for 10 days. In parallel, a protocol for the extraction of anatoxin-a has been optimized beforehand by testing three different solvents on several matrices, with the extraction with 75% methanol + 0.1% formic acid appearing to be the most effective. Anatoxin-a was quantified by high-resolution qTOF mass spectrometry coupled upstream to a UHPLC chromatographic chain. The toxin could not be detected in the livers after 12 h, or in the intestines and muscles after six days. The mean clearance rates of (±)-anatoxin-a calculated after 12 h are above 58%, 100% and 90% for the intestine, the liver and the muscle, respectively. Non-targeted metabolomics investigations performed on the fish liver indicates that the single sub-acute exposure by gavage induces noticeable metabolome dysregulations, including important phospholipid decreases, with an organism recovery period of above 12–24 h. Then, the medaka fish do not appear to accumulate (±)-anatoxin-a and to largely recover after 24 h following a single sub-acute oral liquid exposure at the NOEL.

**Keywords:** anatoxin-a; cyanobacteria; fish toxicity

### 5.11. The Clostridial Neurotoxins: An Expanding Family

Geoffrey Masuyer ^1,2,^* and Pål Stenmark ^2,3^

^1^  Department of Pharmacy and Pharmacology, University of Bath, Bath BA2 7AY, UK
^2^  Department of Biochemistry and Biophysics, Stockholm University, 10691 Stockholm, Sweden
^3^  Department of Experimental Medical Science, Lund University, 22100 Lund, Sweden

* Correspondence: gm283@bath.ac.uk

**Abstract:** The clostridial neurotoxin (CNT) family comprises the tetanus (TeNT) and botulinum neurotoxins (BoNTs), the causative agents of the lethal diseases tetanus and botulism, and represents the most poisonous protein toxins known to man. These extremely potent toxins recognise motor neurons with high affinity and specificity and result in the inhibition of neurotransmission, causing paralysis. Advances in high-throughput genomics from clinical and environmental sources have led to the discovery of several new BoNTs and non-clostridial BoNT-like homologues over the last two years. Our efforts have focused on the biochemical and structural characterisation of these toxins that present novel pharmaceutical and biotechnological potential. Our latest discoveries include BoNT/X, a new BoNT serotype with a unique substrate profile, and PMP1, a clostridial-like neurotoxin that selectively targets anopheline mosquitoes and may provide an innovative, environmentally friendly approach to reduce malaria through anopheline control.

**Keywords:** botulinum; *Clostridium*; neurotoxin

**References**

1. Zhang, S.; Masuyer, G.; Zhang, J.; Shen, Y.; Lundin, D.; Henriksson, L.; Miyashita, S.I.; Martínez-Carranza, M.; Dong, M.; Stenmark, P. Identification and characterization of a novel botulinum neurotoxin. *Nat. Commun.* **2017**, *8*, 14130.
2. Zhang, S.; Lebreton, F.; Mansfield, M.J.; Miyashita, S.I.; Zhang, J.; Schwartzman, J.A.; Tao, L.; Masuyer, G.; Martínez-Carranza, M.; Stenmark, P.; et al. Identification of a botulinum neurotoxin-like toxin in a commensal strain of *Enterococcus faecium*. *Cell Host Microbe* **2018**, *23*, 169–176.
3. Masuyer, G.; Zhang, S.; Barkho, S.; Shen, Y.; Henriksson, L.; Košenina, S.; Dong, M.; Stenmark, P. Structural characterisation of the catalytic domain of botulinum neurotoxin X-high activity and unique substrate specificity. *Sci. Rep.* **2018**, *8*, 4518.
4. Contreras, E.; Masuyer, G.; Qureshi, N.; Chawla, S.; Dhillon, H.S.; Lee, H.L.; Chen, J.; Stenmark, P.; Gill, S.S. A neurotoxin that specifically targets *Anopheles* mosquitoes. *Nat. Commun.* **2019**, *10*, 2869.

### 5.12. Toxicity Assessment of Pinnatoxins: From Molecular Targets to Putative Human Intoxication

César Mattei^1,^*, Nathalie Arnich ^2^, Eric Abadie ^3^, Nicolas Delcourt ^4^, Valérie Fessard ^5^, Jean-Marc Fremy ^2^, Vincent Hort ^6^, Emmeline Lagrange ^7^, Thomas Maignien ^2^, Marie-Bénédicte Peyrat ^2^, Jean-Paul Vernoux ^8^ and Jordi Molgó ^9^

^1^  Mitochondrial and Cardiovascular Pathophysiology, UMR CNRS 6015, Inserm U1083, Angers University, 49100 Angers, France
^2^  Risk Assessment Directorate, ANSES (French Agency for Food, Environmental and Occupational Health and Safety), 94701 Maisons-Alfort, France
^3^  IFREMER (French Research Institute for Exploitation of the Sea), Centre for Marine Biodiversity, Exploitation and Conservation (MARBEC), 34203 Sète, France
^4^  Poison Control Centre, Toulouse-Purpan University Hospital and Toulouse NeuroImaging Centre (ToNIC), 31059 Toulouse, France
^5^  Toxicology of Contaminants Unit, ANSES (French Agency for Food, Environmental and Occupational Health and Safety), 35306 Fougères, France
^6^  Laboratory for Food Safety, ANSES (French Agency for Food, Environmental and Occupational Health and Safety), 94701 Maisons-Alfort, France
^7^  Department of Neurology, Reference Center of Neuromuscular Disease, Grenoble University Hospital, 38000 Grenoble, France
^8^  Research Unit EA 4651 Aliments Bioprocédés Toxicologie Environnements (ABTE), Normandie University, 14000 Caen, France
^9^  CEA (French Alternative Energies and Atomic Energy Commission), Frédéric Joliot Institute for Life Sciences, SIMOPRO (Molecular Engineering of Proteins Unit), 91191 Gif-sur-Yvette, France

* Correspondence: cesar.mattei@univ-angers.fr

**Abstract:** Pinnatoxins (PnTXs) belong to the group of cyclic imines, a large family of emerging neurotoxins, produced by various marine dinoflagellates [1]. The presence of PnTXs has been reported in many marine coastal areas, including France, Spain, New Zealand, Australia, Japan and Canada. Until now, no human poisoning was reported, while these toxins are present in shellfish, including mussels, attesting their ability to be transferred along the food chain. Several studies have shown that PnTXs are potent competitive antagonists of nicotinic acetylcholine receptors [2,3]: their binding induces a reversible blocking of the ion flux carried by these ionotropic receptors. As a result, PnTXs are able to alter central and peripheral cholinergic networks in vivo. In mouse models, exposure to PnTXs induced neurologic symptoms few minutes after ingestion, including reduced mobility, muscular paralysis, convulsive episodes, jumps and respiratory troubles, eventually leading to death at higher doses. At sublethal doses, animals recovered, with no apparent sequelae. We extrapolated human symptoms that may be observed in cases of shellfish intoxication, from what is currently known of the PnTX mode of action in vitro and in vivo. As there is currently no regulatory threshold for PnTXs, a risk assessment work has been carried out on PnTX G, the mostly detected PnTX in shellfish in France. A provisional acute benchmark value for human oral exposure was proposed, based on the dose-dependent symptoms observed in mice^4^. For a large portion size of shellfish consumption, the concentration of PnTX G in shellfish not expected to induce adverse effects in humans was derived and may be used as a basis for the establishment of a regulatory limit in shellfish.

**Keywords:** health risk assessment; muscle paralysis; nicotinic receptors; pinnatoxins; *Vulcanodinium rugosum*

**References**

1. Delcourt, N.; Lagrange, E.; Abadie, E.; Fessard, V.; Frémy, J.M.; Vernoux, J.P.; Peyrat, M.B.; Maignien, T.; Arnich, N.; Molgó, J.; et al. Pinnatoxins’ deleterious effects on cholinergic networks: from experimental models to human health. *Mar. Drugs* **2019**, *17*, 425.
2. Aráoz, R.; Servent, D.; Molgó, J.; Iorga, B.I.; Fruchart-Gaillard, C.; Benoit, E.; Gu, Z.; Stivala, C.; Zakarian, A. Total synthesis of pinnatoxins A and G and revision of the mode of action of pinnatoxin A. *J. Am. Chem. Soc.* **2011**, *133*, 10499–10511.
3. Benoit, E.; Couesnon, A.; Lindovsky, J.; Iorga, B.I.; Aráoz, R.; Servent, D.; Zakarian, A.; Molgó, J. Synthetic Pinnatoxins A and G reversibly block mouse skeletal neuromuscular transmission in vivo and in vitro. *Mar. Drugs* **2019**, *17*, 306.
4. Arnich, N.; Abadie, E.; Delcourt, N.; Fessard, V.; Fremy, J.-M.; Hort, V.; Lagrange, E.; Maignien, T.; Molgó, J.; Peyrat, M.-B.; et al. Health risk assessment related to pinnatoxins in French shellfish. *Toxicon* **2019**, submitted.

### 5.13. Pinnatoxins A and G Potently and Reversibly Block Transmission at the Skeletal Neuromuscular Junction In Vivo and In Vitro

Evelyne Benoit ^1,2^, Aurélie Couesnon ^2^, Jiri Lindovsky ^2^, Bogdan I. Iorga ^3^, Denis Servent ^1^, Armen Zakarian ^4^ and Jordi Molgó^1,2,^*

^1^  CEA, Institut des Sciences du Vivant Frédéric Joliot, Service d’Ingénierie Moléculaire des Protéines, CEA de Saclay, Université Paris-Saclay, 91191 Gif-sur-Yvette, France
^2^  CNRS, Institut des Neurosciences Paris-Saclay, UMR 9197 CNRS/Université Paris-Sud, 91198 Gif-sur-Yvette, France
^3^  CNRS, Institut de Chimie des Substances Naturelles, UPR 2301, Labex LERMIT, 91198 Gif-sur-Yvette, France
^4^  Department of Chemistry and Biochemistry, University of California Santa Barbara, Santa Barbara, CA 93106-9510, USA

* Correspondence: jordi.molgo@cea.fr

**Abstract:** The dinoflagellate *Vulcanodinium rugosum,* first isolated from Ingril, a French Mediterranean lagoon, is known to produce the pinnatoxins (PnTXs) and the portimines. PnTXs (A-H) constitute an emerging family of phycotoxins belonging to the cyclic imine group [1,2]. Interest has been focused on these fast-acting, highly potent toxins because they are widely found in contaminated shellfish. Despite their highly complex molecular structure, PnTXs have been chemically synthetized by the Zakarian group, and demonstrated to act on various nicotinic acetylcholine receptors (nAChRs) [3,4]. To the best of our knowledge, neither PnTX-A nor PnTX-G and analogs, obtained by chemical synthesis with high degree of purity (>98%), have been studied in vivo or in vitro on adult mouse and isolated nerve–muscle preparations expressing the mature muscle-type (α1)_2_β1εδ nAChR. Our results show that PnTX-A and PnTX-G acted on the neuromuscular system of anesthetized mice and blocked the compound muscle action potential (CMAP) in a dose-and time-dependent manner with similar ID_50_ values (dose required to block 50% of the CMAP), as determined using an in vivo, minimally invasive electrophysiological method. The decrease of CMAP induced by both toxins in vivo was reversible within 6–8 h. PnTX-A and PnTX-G, applied to isolated *extensor digitorum longus* (EDL) nerve–muscle preparations, blocked reversibly isometric twitches evoked by nerve stimulation. Both toxins exerted no direct action on the contractile machinery of muscle fibers, as revealed by direct muscle stimulation. In addition, PnTX-A and PnTX-G blocked synaptic transmission at mouse neuromuscular junctions. PnTX-A aminoketone analog (containing an open form of the imine ring) [4] had no effect on neuromuscular transmission. These results indicate the importance of the cyclic imine for interacting with adult muscle-type nAChR.

**Keywords:** compound muscle action potential; cyclic imine; emerging toxin; marine phycotoxin; mouse neuromuscular system; pinnatoxin; synaptic potential

**Funding:** Supported in part by NIH/NIGMS grant GM R01-077379 and by Interreg Atlantic program project ALERTOX-NET-EAPA_317/2016).

**References**

1. Stivala, C.E.; Benoit, E.; Aráoz, R.; Servent, D.; Novikov, A.; Molgó, J.; Zakarian, A. Synthesis and biology of cyclic imine toxins, an emerging class of potent, globally distributed marine toxins. *Nat. Prod. Rep.* **2015**, *32*, 411–435.
2. Molgó, J.; Marchot, P.; Aráoz, R.; Benoit, E.; Iorga, B.I.; Zakarian, A.; Taylor, P.; Bourne, Y.; Servent, D. Cyclic imine toxins from dinoflagellates: A growing family of potent antagonists of the nicotinic acetylcholine receptors. *J. Neurochem.* **2017**, *142* (Suppl. 2), 41–51.
3. Aráoz, R.; Servent, D.; Molgó, J.; Iorga, B.I.; Fruchart-Gaillard, C.; Benoit, E.; Gu, Z.; Stivala, C.; Zakarian, A. Total synthesis of pinnatoxins A and G and revision of the mode of action of pinnatoxin A. *J. Am. Chem. Soc.* **2011**, *133*, 10499–10511.
4. Bourne, Y.; Sulzenbacher, G.; Radić, Z.; Aráoz, R.; Reynaud, M.; Benoit, E.; Zakarian, A.; Servent, D.; Molgó, J.; Taylor, P.; et al. Marine macrocyclic imines, pinnatoxins A and G: Structural determinants and functional properties to distinguish neuronal α7 from muscle α12β1γδ nAChRs. *Structure* **2015**, *23*, 1106–1115.

### 5.14. Improved In Vitro Assays Using Human Cell Lines of Neuronal Linage and Primary Neuronal Cells for Testing of BoNT Toxins and Antitoxins

Christine Rasetti-Escargueil^1,^* **, Marc Garcia Riera ^1^, Yagmur Derman ^3^, Christian Lévèque ^4^, Emmanuel Lemichez ^1,2^ and Michel R. Popoff ^1^**

^1^  Institut Pasteur, Microbiology Department, Bacterial Toxins Unit, 25 rue du Docteur Roux, 75015 Paris, France
^2^  Department of Infectious diseases and Microbiology, Université Paris Diderot, Sorbonne Paris Cité, 75205 Paris, France
^3^  Department of Food Hygiene and Environmental Health, Faculty of Veterinary Medicine, University of Helsinki, P.O. Box 66, 00014 Helsinki, Sweden
^4^  Inserm UMR 1072, Aix-Marseille Université, 13344 Marseille CEDEX 15, France

* Correspondence: christine.rasetti-escargueil@pasteur.fr

**Abstract:** Background: There is a pressing need to develop and validate European testing capabilities to deal with the threat of biological toxins. Moreover, low doses of the toxin are increasingly used as licensed drugs for the treatment of a variety of medical disorders such as movement disorders, dystonia, hyperhidrosis, essential tremor, chronic pain, migraine, overactive bladder, some forms of depression and for cosmetic purposes. A wide range of available in vitro analytical tools is currently employed during the EuroBiotox project, to harmonize testing capabilities as well as reduce animal use. Methods: This study utilized procedures generating sustainable mature neurons as well as primary neuronal cells. The detection of neurotoxin activity was achieved by monitoring BoNT/A- and BoNT/E-induced SNAP-25 cleavage through quantification of intracellular proteins in parallel to in vitro endopeptidase assay. The multi-electrode array (MEA) technology established previously was assessed as a potential biosensor platform. Results: A robust differentiation protocol for human neuronal cell line, together with suitable read outs, provides an opportunity for the development of next generation bioassays. A dose-dependent increase in BoNT/A-induced SNAP cleavage was observed after the intoxication of neurons. The in vitro endopeptidase immuno-assay provided a highly reproducible detection of BoNT/A and BoNT/E, using specific SNAP25 cleavage site detecting antibodies. Bath application of BoNT/A lead to the silencing of neuronal network activity. Conclusions: The evaluation of currently available in vitro methods as a replacement for animal tests for botulinum neurotoxin (BoNT) detection and titration, able to entirely replace animal models, will have a great impact on public health and the economy. If applied globally by manufactures for therapeutic product control, this will lead to unprecedented animal use reduction.

**Keywords:** antitoxin; botulinum neurotoxin; in vitro assay; neuronal cell

### 5.15. Bioactive Natural Peptides Candidate for Outstanding Therapies: How to Deal with ADME Behaviors and Pharmacokinetic Parameters?

Marc Ravatin^1,2,^*, Grazyna Faure ^2^, Jean-Marie Chambard ^1^ and Pierre-Jean Corringer ^2^

^1^  Sanofi, Integrated Drug Discovery–High Content Screening and Sample Management, 13 quai Jules Guesde, 94403 Vitry-sur-Seine, France
^2^  Institut Pasteur, Channels/Receptors Unit, CNRS UMR3571, 25 rue du Docteur Roux, 75015 Paris, France

* Correspondence: marc.ravatin@pasteur.fr

**Abstract:** The diversity of natural peptides, including toxins, makes them a broad source of chemical bioactive scaffolds. Indeed, they are naturally designed to exert their bioactivities on specific targets. Because of this high specificity, many of them have few off-target effects. These natural properties are greatly appreciated by scientists working on R&D programs. However, beyond these properties, the weaknesses of natural peptides can be found in their biostability and bioavailability. These two pharmacological parameters can explain these disheartening statistics: for every 350 peptide candidates submitted to the FDA, only five will go up to phase 3 and then only two will be released on the market. Nevertheless, a lot of chemical strategies are widely used to improve the pharmacokinetic properties of peptide candidates for clinical trials. Some chemical modifications are well known, such as cyclisation, the use of non-natural amino acids and lipidation of amino acids. This poster discusses chemical optimization strategies to (1) extend the half-life of peptides and small toxins through enzyme resistance and the reduction of kidney clearance, (2) enhance the cell-penetration properties of peptides and finally (3) optimize some toxins and toxin mimetics by the techniques used for R&D programs and clinical trials.

**Keywords:** ADME parameter; natural peptide; peptidic therapeutic candidate; pharmacokinetic parameter

### 5.16. The First Venom-Derived Melanocortin 1 Receptor Agonists

Steve Reynaud ^1,2,^*, Justyna Ciolek ^2^, Hervé Meudal ^3^, Céline Landon ^3^, Agnès Delmas ^3^, Annette G. Beck-Sickinger ^4^, Karin Morl ^4^, Julia Boeri ^2^, Denis Servent ^2^, Ricardo C. Rodriguez De La Vega ^5^, Gilles Mourier ^2^ and Nicolas Gilles ^2^

^1^  Université Paris-Sud, 15 rue Georges Clemenceau, 91400 Orsay, France
^2^  Service d’Ingénierie Moléculaire des Protéines (SIMOPRO), CEA, Université Paris-Saclay, 91191 Gif-sur-Yvette, France
^3^  Laboratoire de RMN, Centre de Biophysique Moléculaire, rue Charles Sadron, 45071 Orléans CEDEX 02, France
^4^  Institute of Biochemistry, Faculty of Life Sciences, Leipzig University, 4107 Leipzig, Germany
^5^  Écologie Systématique Évolution, Université Paris-Sud, AgroParisTech, CNRS, Université Paris-Saclay, 91400 Orsay, France

* Correspondence: steve-reynaud@orange.fr

**Abstract:** The melanocortin receptors (MCRs) are a G protein-coupled receptors (GPCRs) family of five different subtypes. Each of them displays a particular physiological function and two, the MC1R and the MC4R, are of high therapeutic interest. For instance, loss-of-function mutations on subtype 4 (MC4R) are the primary cause of monogenic obesity [1], making it a therapeutic target in eating disorders. Subtype 1 (MC1R) plays a crucial protective role against melanoma. It elicits eumelanin production, a dark, UV radiation absorbing, photo-protective pigment [2] and promotes the nucleotide excision repair of DNA damaged by UV radiation [3]. There is an urgent need for the development of innovative ligands, to better understand MCR functions and to generate new medicines. Following the screening of a synthetic animal toxin bank on MC4R, we obtained an incredible hit rate of 9%. Among the identified hits, two toxins were particularly studied: N-TRTX-Preg1a and N-BUTX-Ptr1a. Both toxins belong to two well-known toxin families, inhibitory cystine knot (ICK) motif and a short scorpion-CSαβ-like structure, respectively. The two scaffolds are primary known to block ion channels, yet neither N-TRTX-Preg1a nor N-BUTX-Ptr1a affect ion channel activity. Phylogenetically, these two toxins form new groups within their respective families. N-TRTX-Preg1a and N-BUTX-Ptr1a bind to the melanocortin receptors with low micromolar affinities and activate the MC1R/Gs pathway without the common pharmacophore previously known to be essential for receptor activation [4,5]. Thus, they are not only the first animal toxins active on MCRs but also the first MC1R peptidic agonists, unrelated to the natural agonists. This may aid the generation of ligands with improved affinity and selectivity in the development of new MC1R agonists with applications in the treatment and/or prevention of melanoma.

**Keywords:** G protein-coupled receptors; melanocortin receptors; melanoma; toxin

**References**

1. The Portal for Rare Diseases and Orphan Drugs. Available online: https://www.orpha.net (accessed on 1 January 2020).
2. Kobayashi, N.; Nakagawa, A.; Muramatsu, T.; Yamashina, Y.; Shirai, T.; Hashimoto, M.W.; Ishigaki, Y.; Ohnishi, T.; Mori, T. Supranuclear melanin caps reduce ultraviolet induced DNA photoproducts in human epidermis. *J. Investig. Dermatol.* **1998**, *110*, 806–810.
3. Kadekaro, A.L.; Leachman, S.; Kavanagh, R.J.; Swope, V.; Cassidy, P.; Supp, D.; Sartor, M.; Schwemberger, S.; Babcock, G.; Wakamatsu, K.; et al. Melanocortin 1 receptor genotype: an important determinant of the damage response of melanocytes to ultraviolet radiation. *FASEB J.* **2010**, *24*, 3850–3860.
4. Hruby, V.J.; Wilkes, B.C.; Hadley, M.E.; Al-Obeidi, F.; Sawyer, T.K.; Staples, D.J.; DeVaux, A.E.; Dym, O.; Castrucci, A.M.D.L. Alpha-Melanotropin: the minimal active sequence in the frog skin bioassay. *J. Med. Chem*. **1987**, *30*, 2126–2130.
5. Singh, A.; Haslach, E.M.; Haskell-Luevano, C. Structure-activity relationships (SAR) of melanocortin and agouti-related (AGRP) peptides. *Adv. Exp. Med. Biol.* **2010**, *681*, 1–18.

### 5.17. Bi-Component Pore-Forming Toxins of *Staphylococcus aureus* Modulate Specifically the Membrane ions Flux in Human Neutrophils

Leila Staali *^,^ **^†^ and Didier A. Colin**

Institut de Bactériologie de la Faculté de Médecine, Université Louis Pasteur, 67000 Strasbourg, France

* Correspondence: lstaali1@yahoo.com

† Present address: Département de Biotechnologie, Faculté des Sciences de la Nature et de la Vie, Université d’Oran 1 Ahmed Ben Bella, 31000 Oran, Algeria.

**Abstract:***Staphylococcus aureus* (*S. aureus*) is a major bacterial pathogen that causes significant infectious diseases by producing several virulence factors. Among these, one can find bi-component pore-forming toxins (PFTs), such as the Panton and Valentin leukocidin (PVL) and γ-hemolysin, which act in synergy on the membrane of host cells. In this study, the molecular mechanisms by which these leucotoxins modulate the membrane ions flux in human polymorphonuclear neutrophils were investigated using spectrofluorometry techniques and specific molecular probes. Our data clearly demonstrate that following the specific binding of these leucotoxins on receptors, a strong activation of target cells induces rapid changes in intracellular free calcium levels ([Ca^2+^]). Interestingly, this phenomenon was associated with intracellular signal transduction events involved in the immune host responses against leucotoxin-mediated cellular damage. In human neutrophils, the massive changes in [Ca^2+^] resulted from the opening of different types of pre-existing Ca^2+^-channels. This event was rapidly followed by an additional effect of PFTs, which induces the activation of specific monovalent ions channels. The later event clearly argues with and confirms our data, suggesting the specificity of the pores formed independently by all leucotoxin couples tested. Finally, we conclude that mechanisms involved in *S. aureus* resistance during staphylococcal infection could be linked and/or regulated by the intracellular changes in [Ca^2+^]. Moreover, the diversity in the leucotoxin and γ–hemolysin effects observed could explain the diversity in effects within *S. aureus* clinical isolates.

**Keywords:** Ca^2+^-channel; γ-hemolysin; Panton and Valentin leukocidin; polymorphonuclear neutrophil; *S. aureus*; spectrofluorometry

### 5.18. Optimisation of a Broad-Spectrum Inhibitor of Toxins Selected from a CNF1-Induced Rac1 Modulator

Florian Ville^1,2,^*, Nassim Mahtal ^1,2^, Victor Kreis ^1^, Léa Swistak ^3^, Amel Mettouchi ^3^, Christine Rasetti-Escargueil ^3^, Jean-Christophe Cintrat ^2^, Julien Barbier ^1^, Michel R. Popoff ^3^, Emmanuel Lemichez ^3^ and Daniel Gillet ^1^

^1^  Service d’Ingénierie Moléculaire des Protéines (SIMOPRO), Bât 152, 91191 Gif-sur-Yvette, France
^2^  Service de Chimie Bioorganique et de Marquage (SCBM), Bât 547, 91191 Gif-sur-Yvette, France
^3^  Institut Pasteur, Unité de Toxines Bactériennes, 25–28 rue du Docteur Roux, 75015 Paris, France

* Correspondence: florian.ville@cea.fr

**Abstract:** Bacterial toxins are virulence factors responsible for human diseases and potential bioterror weapons. Through their ability to reach the cytosol, they exert their enzymatic effects and disrupt the normal metabolism of host cells, producing deleterious effects and generally leading to their death. This study aims to develop a drug candidate capable of blocking the action of a broad range of bacterial toxins. A High Throughput Screening identified a compound with broad-spectrum inhibitory properties against many bacterial toxins (LT, BoNT/A, DT, CNF1, TcdB, Stx), therefore constituting a good starting point for the development of a drug candidate by medicinal chemistry. This chemical optimization, based on structure activity relationship studies, is currently underway. In order to identify the chemical groups essential for bioactivity and to get more potent molecules, thirty-seven analogs were synthesized so far, and their bioactivity as well as toxicity was evaluated in vitro in cellular assays. The results obtained open up prospects to improve the activity of the parent molecule.

**Keywords:** bacterial toxin; broad toxin inhibitor; drug discovery; medicinal chemistry; SAR study

### 5.19. Evaluation of the Toxicity of an Insecticide Mixture Widely Used in the Jijel Wilaya on a Biological Model: The Snail *Helix aspersa*

Mohamed Fateh Zouaghi *, Islerm Boudoucha and Ibrahim Rekaik

Département des Sciences de l’Environnement et des Sciences Agronomiques, Université de Jijel, Faculté des Sciences de la Nature et de la Vie, 18000 Jijel, Algeria

* Correspondence: fatehmilan@hotmail.fr

**Abstract:** In this study, we were interested in evaluating the impact of a mixture of two neonicotinoid insecticides and their effects on a bioaccumulator organism and bioindicator of pollution, *Helix aspersa*. This is a subchronic toxicity study (21 days). Insecticide toxicity was determined in *H. aspersa* snail using laboratory biotest in animals exposed to increasing concentrations of insecticide mixture. Our results highlight physiological disturbances concerning the shell weight and diameter of treated snails. At the same time, the metabolic changes indicate a disruption in the protein content and a decrease in the level of carbohydrates in the hepatopancreas and kidneys. Our results also show the existence of an induction of catalase activity, one of the cellular defense mechanisms against the presence of insecticides in the two targeted organs.

**Keywords:** bioaccumulator; pollution; snail

## 6. Acknowledgments

We warmly acknowledge the contribution of all those people who work daily at ensuring the national and international shinning of the French Society of Toxinology (SFET), and those who made the 26th Meeting on Toxinology a success. We also offer special thanks to our sponsors who, again this year, supported our meeting (their logos are shown below).